# The influence of COVID-19 on colorectal cancer was investigated using bioinformatics and systems biology techniques

**DOI:** 10.3389/fmed.2023.1169562

**Published:** 2023-06-30

**Authors:** Yujia Song, Tengda Huang, Hongyuan Pan, Ao Du, Tian Wu, Jiang Lan, Xinyi Zhou, Yue Lv, Shuai Xue, Kefei Yuan

**Affiliations:** ^1^Division of Liver Surgery, Department of General Surgery and Laboratory of Liver Surgery, and State Key Laboratory of Biotherapy and Collaborative Innovation Center of Biotherapy, West China Hospital, Sichuan University, Chengdu, China; ^2^State Key Laboratory for Conservation and Utilization of Subtropical Agro-Bioresources, College of Animal Science and Technology, Guangxi University, Nanning, China

**Keywords:** COVID-19, colorectal cancer, differentially expressed genes, gene ontology, protein–protein interaction, hub gene, drug molecule

## Abstract

**Introduction:**

Coronavirus disease 2019 (COVID-19) is a global pandemic and highly contagious, posing a serious threat to human health. Colorectal cancer (CRC) is a risk factor for COVID-19 infection. Therefore, it is vital to investigate the intrinsic link between these two diseases.

**Methods:**

In this work, bioinformatics and systems biology techniques were used to detect the mutual pathways, molecular biomarkers, and potential drugs between COVID-19 and CRC.

**Results:**

A total of 161 common differentially expressed genes (DEGs) were identified based on the RNA sequencing datasets of the two diseases. Functional analysis was performed using ontology keywords, and pathway analysis was also performed. The common DEGs were further utilized to create a protein-protein interaction (PPI) network and to identify hub genes and key modules. The datasets revealed transcription factors-gene interactions, co-regulatory networks with DEGs-miRNAs of common DEGs, and predicted possible drugs as well. The ten predicted drugs include troglitazone, estradiol, progesterone, calcitriol, genistein, dexamethasone, lucanthone, resveratrol, retinoic acid, phorbol 12-myristate 13-acetate, some of which have been investigated as potential CRC and COVID-19 therapies.

**Discussion:**

By clarifying the relationship between COVID-19 and CRC, we hope to provide novel clues and promising therapeutic drugs to treat these two illnesses.

## 1. Introduction

Severe acute respiratory syndrome coronavirus 2 (SARS-CoV-2) is a betacoronavirus, belonging to the subgenus *Sarbecovirus*, which is transmitted primarily through the respiratory tract and is highly potent and infectious (1). SARS-CoV-2 has unique features compared with previously known coronavirus genomes, including an optimum affinity for the angiotensin-converting enzyme 2 (ACE2) receptor and a polybasic cleavage site at the S1/S2 spike junction, which determines infectivity and host range (2). In addition, it was shown that SARS-CoV-2 also enters host cells with the primary or auxiliary help of host proteases transmembrane protease serine 2 (TMPRSS2) and FURIN (3), glucose-regulating protein 78 (GRP78) receptor (4), dipeptidyl peptidase 4 (DPP4), (5), cluster of differentiation 147 (CD147) transmembrane protein (6), tyrosine-protein kinase receptor UFO (AXL) (7), phosphatidylinositol 3-phosphate 5-kinase (PIKfyve), two pore channel subtype 2 (TPC2) and cathepsin L (8). The respiratory illness coronavirus disease 2019 (COVID-19) is caused by SARS-CoV-2 (9). Infection with SARS-CoV-2 can stimulate both innate and adaptive immune responses, whereas an uncontrolled inflammatory innate response and a defective adaptive immune response may lead to detrimentally local and systemic tissue damage (9, 10). Patients with COVID-19 frequently experience symptoms such as fever, coughing, vomiting, diarrhea, abdominal pain, shortness of breath, and severe cases that can develop to acute respiratory distress syndrome (ARDS), pneumonia, and multi-organ failure (10, 11). Since its emergence, COVID-19 has continued to affect many parts of the world and threaten millions of lives worldwide (12). Wang et al. have shown that the case fatality rate of COVID-19 patients ranges from 2 to 3% (13). Although the lung is the primary target of coronavirus infection, the digestive system has also been identified to have ACE2, the SARS-CoV-2 functional receptor, suggesting that in addition to the respiratory system, COVID-19 can also infect people through this system (1, 14).

Several studies have been published on the effects of COVID-19 on cancer patients (15–17). A previous study has shown that cancer patients are more vulnerable to infection than individuals without cancer due to the systemic immunosuppressive state created by cancer and anticancer treatment, such as chemotherapy or surgery (17). Yeo et al. further found that cancer was linked to a 2.84-fold greater risk of severe disease in COVID-19 patients and a 2.60-fold increased risk of mortality (18). Colorectal cancer (CRC) is the world’s second-largest cause of cancer death and the third most common malignancy (19). A subgroup analysis based on place of origin showed that Chinese CRC patients had a significant risk of infection, with the global burden of COVID-19 infection in CRC patients being 45.1% (20). According to Burgueño et al., the ileum, colon, and intestinal epithelial cells express ACE2 and TMPRSS2, which permits COVID-19 access into colon cells (21). Immunosuppression, malignancy, and anticancer therapy may play a role in the increased COVID-19 susceptibility in CRC (22). Therefore, it is crucial to explore how COVID-19 influences CRC patients and to search for possible therapeutic agents for CRC patients with COVID-19.

**Figure 1:**
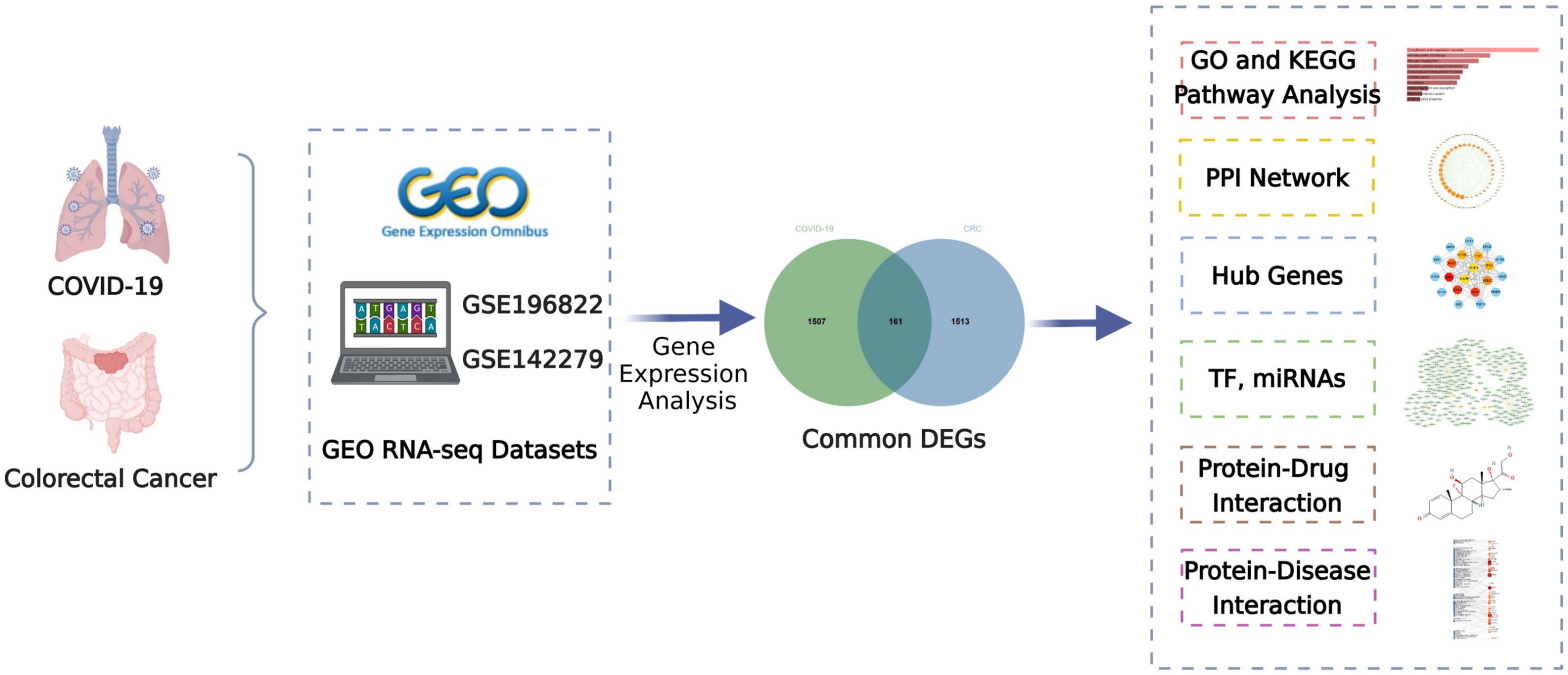
Overall flowchart of this research.

This study attempts to search the shared biological pathways and possible drugs between CRC and COVID-19 by bioinformatics and systems biology techniques. The COVID-19 datasets (GSE196822) and CRC datasets (GSE142279) were from the Gene Expression Omnibus (GEO) database, respectively. Initially, COVID-19 and CRC differentially expressed genes (DEGs) were found, and then common DEGs of the two illnesses were discovered. The obtained common DEGs served as the foundation for subsequent research. Then, employing common DEGs, enrichment analysis and pathway analysis were used to better understand the biological process of gene expression. Protein–protein interaction (PPI) networks were also utilized to gather hub genes based on common DEGs. Transcriptional regulators and miRNAs were then identified using common DEGs. Finally, prospective drugs are offered, as well as gene-disease associations. Figure 1 depicts the overall workflow of this study.

## 2. Materials and methods

### 2.1. Data collection

To determine the common gene interrelationship between COVID-19 and CRC, we downloaded RNA-seq datasets of COVID-19 and CRC from the GEO database of the National Center for Biotechnology Information (NCBI)^1^ (23). The GEO accession ID of COVID-19 was GSE196822, which included RNA-seq profiling from 9 uninfected samples and 34 infected samples using the high throughput sequencing Illumina HiSeq 4,000 (*Homo sapiens*) platform (24). Similarly, the sequencing of CRC (GSE142279) was gained by high throughput sequencing HiSeq X Ten (*Homo sapiens*) platform from 20 pairs of colorectal tumors with matched adjacent normal colorectal tissue.

### 2.2. Determination of differentially expressed genes and mutual DEGs among COVID-19 and CRC

A gene is believed to be expressed differently when there is a transcription level statistically significant difference between distinct test conditions (25). To extract DEGs for datasets GSE196822, we utilized the “DESeq2” package (26) of the R program (version 4.2.2) with False Discover Rate (FDR) < 0.05 and |log_2_ Fold Change| > 1. The dataset GSE142279 was retrieved by the “limma” package (27) and the criteria for considering the difference in expression as FDR < 0.05 and |log_2_ Fold Change| > 1. Common DEGs of the two datasets were obtained from the online Venn analysis program Jvenn^2^ (28).

### 2.3. Gene ontology and pathway enrichment analysis

The Gene Ontology is a globally standardized gene functional classification system with three ontologies: molecular function, cellular component, and biological process. The primary biological functions of DEGs were identified using this analysis (29). By comparing DEGs to the whole genome background, pathway enrichment analysis showed significantly enriched metabolic or signal transduction pathways. Pathway databases included KEGG (Kyoto Encyclopedia of Genes and Genomes), WikiPathways, Reactome, and BioCarta. In this research, we utilized the online gene-set enrichment tool EnrichR^3^ to investigate the biological processes and signaling pathways linked to common DEGs (30). To quantify the most important functional items and pathways, *p*-values of 0.05 was employed as standardized metrics.

### 2.4. PPI network analysis and hub gene prediction

PPI is an important part of the cellular biochemical response network and can be used to map the functional and structural knowledge of cellular protein networks (31). STRING^4^ (version 11.5) was utilized to perform the key assessment of protein–protein interactions based on the common DEGs. With a combined score larger than 0.4, the PPI network was created and visualized using Cytoscape (v.3.9.1). The most entangled nodes in the PPI network are referred to as hub genes. To rank and analyze the major nodes in the PPI network module and identify the key genes, we used the Cytoscape plugin cytoHubba. The top ten hub genes in the PPI network were chosen using the Maximal Clique Centrality (MCC) method (32).

### 2.5. Identification of transcription factors and MiRNAs

TFs are proteins that bind to specific genes and regulate the transcription rate of genetic information. They built a sophisticated system that regulated genome expression and contributed significantly to molecular understanding (33). The DEGs-TFs interaction network was established using the JASPAR database from the online website NetworkAnalyst. NetworkAnalyst was developed in response to the crucial requirement for analyzing gene expression data within the framework of PPI networks to obtain information on biological mechanisms, functions, and interpretations (34). JASPAR is the most complete database of TF-DNA binding site motifs available to the public (35). MiRNAs that target gene interactions were incorporated to identify miRNAs with the potential to bind to gene transcripts and so negatively impact protein production. The miRTarBase, based on NetworkAnalyst and focused on topological analysis, was utilized to identify DEG-miRNA interaction networks. MiRTarBase is the most famous overall miRNA-target interaction database and an important database for evaluating the validity of miRNA-target gene interaction experiments (36). Cytoscape was used to display the TF-gene and miRNA-gene interaction networks.

### 2.6. Evaluation of potential drugs

Predicting protein-drug interactions (PDI) or identifying pharmacological molecules is an essential part of this research. The Drug Signatures database (DSigDB) database was employed to identify the common DEGs by accessing the enrichment of Enrichr under the Diseases/Drugs function on the online website. DSigDB is a gene set resource for identifying targeted drugs associated with DEGs (37). Further screening of significant drug molecules was performed based on the *p*-value.

### 2.7. Gene-disease association analysis

DisGeNET,^5^ one of the biggest and most thorough databases of human gene-disease connections, integrates and standardizes data on disease-associated genes and variants from various sources while also providing a variety of biomedical aspects of the disease (38). The NetworkAnalyst platform was used to construct a gene-disease interaction network to discover diseases and chronic complications linked to common DEGs (39).

## 3. Results

### 3.1. Identification of DEGs and common DEGs between COVID-19 and CRC

**Figure 2:**
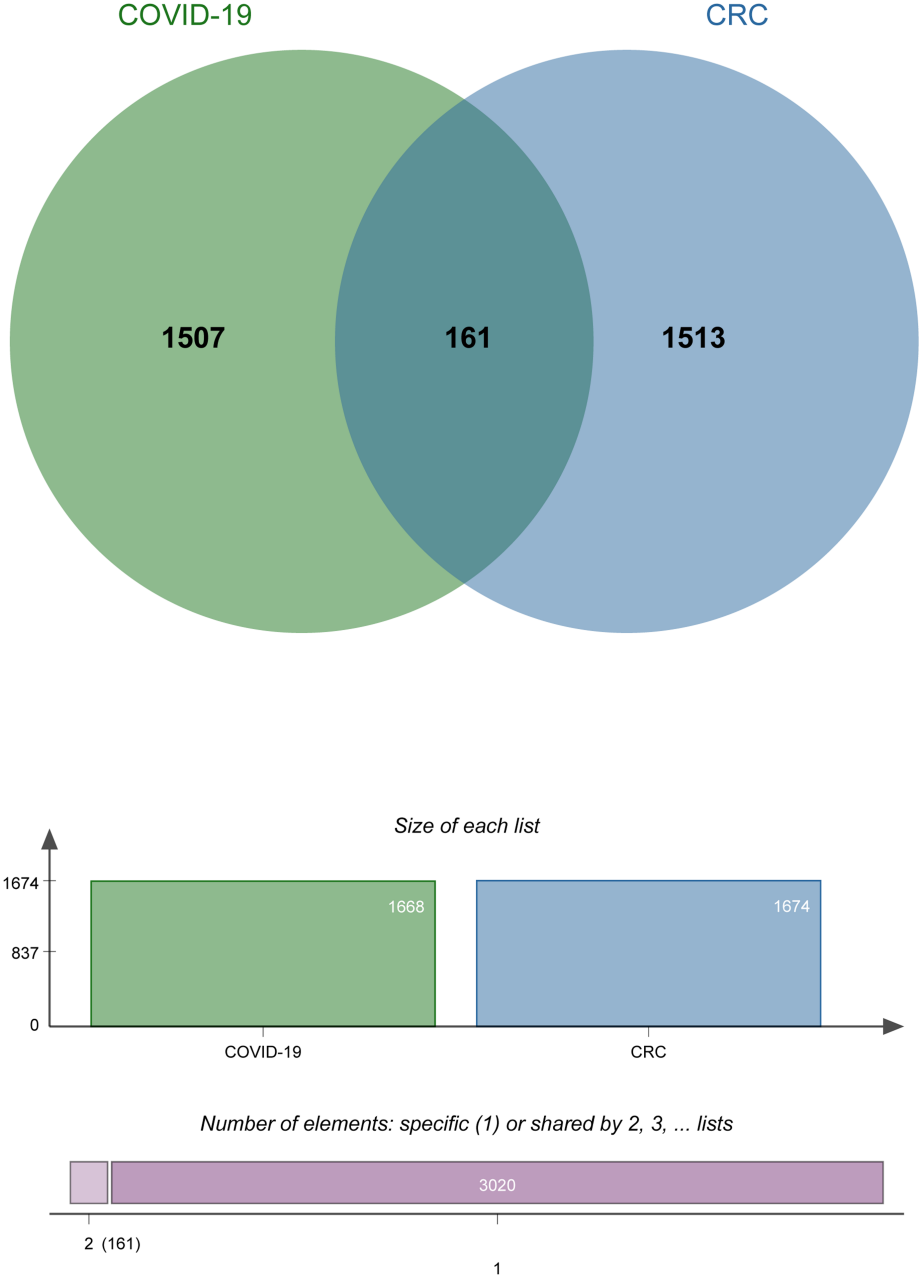
The Venn diagram of the DEGs shared by COVID-19 and CRC.

To investigate the interrelationship and impact between COVID-19 and CRC, we collected corresponding human RNA-seq datasets, respectively. We identified a total of 1,668 differentially expressed genes between COVID-19 patients and healthy controls, 839 of which showed up-regulated and 829 down-regulated. At the same time, 1,674 in total DEGs were found between colorectal tumors and normal colorectal tissues in CRC patients, including 751 up-regulated DEGs and 923 down-regulated DEGs (Table 1). We discovered 161 common DEGs from COVID-19 and CRC datasets through cross-comparison analysis using the Jvenn (28), a reputable Venn analysis platform, suggesting that they may be related to the biological link between these two conditions (Figure 2). The results of the differential expression study showed that COVID-19 and CRC interacted and shared certain molecular similarities.

**Table 1:** Overview of the datasets used in this study along with their geo-features and quantitative measurements.

Disease name	GEO accession	GEO platform	Total DEGs count	Up regulated DEGs count	Down regulated DEGs count
COVID-19	GSE196822	GPL20301	1,668	839	829
CRC	GSE142279	GPL20795	1,674	751	923

### 3.2. Gene ontology and pathway enrichment analysis

**Figure 3:**
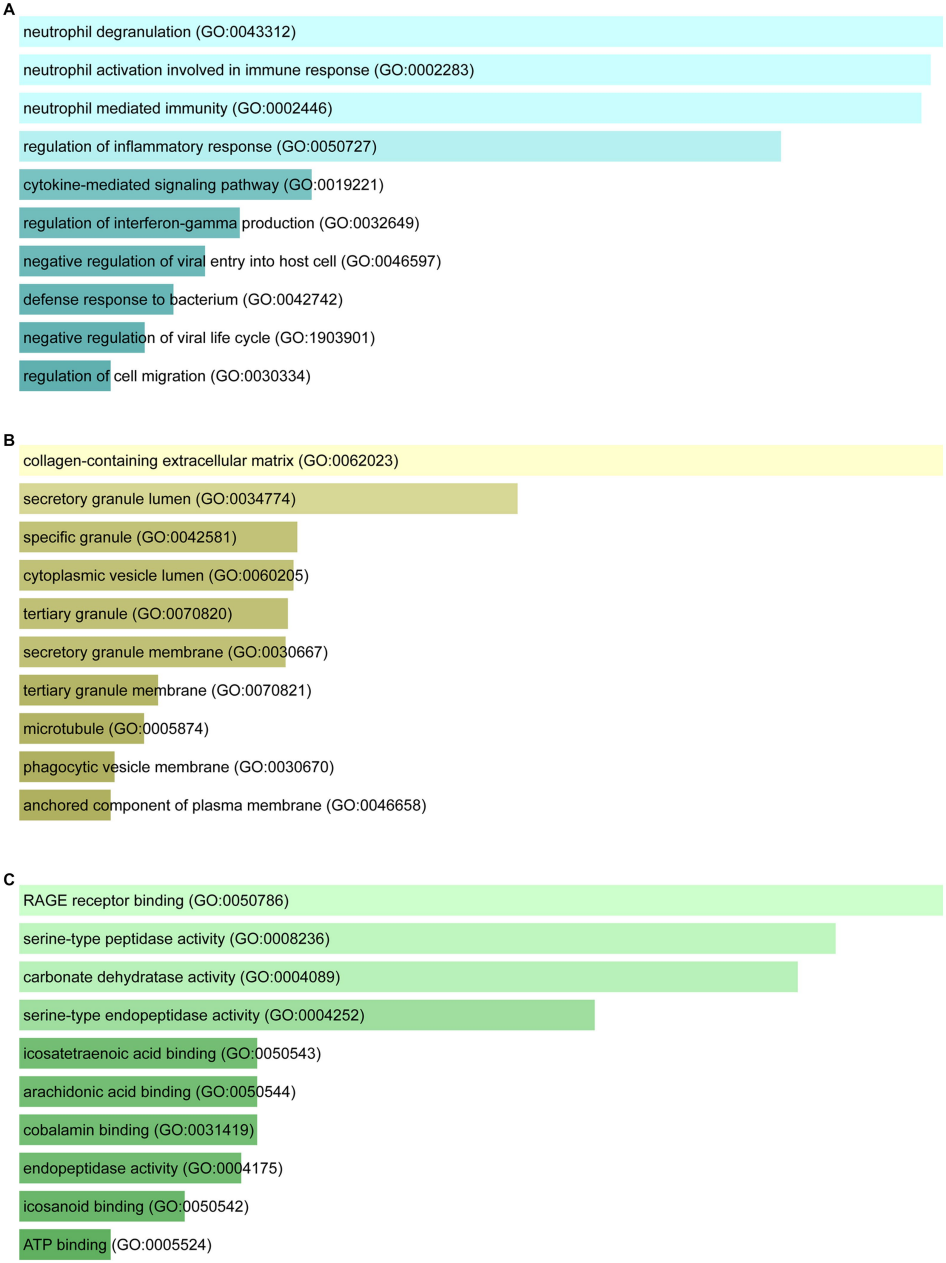
Ontological analysis of shared DEGs: **(A)** Biological processes, **(B)** cellular components, and **(C)** molecular functions.

**Figure 4:**
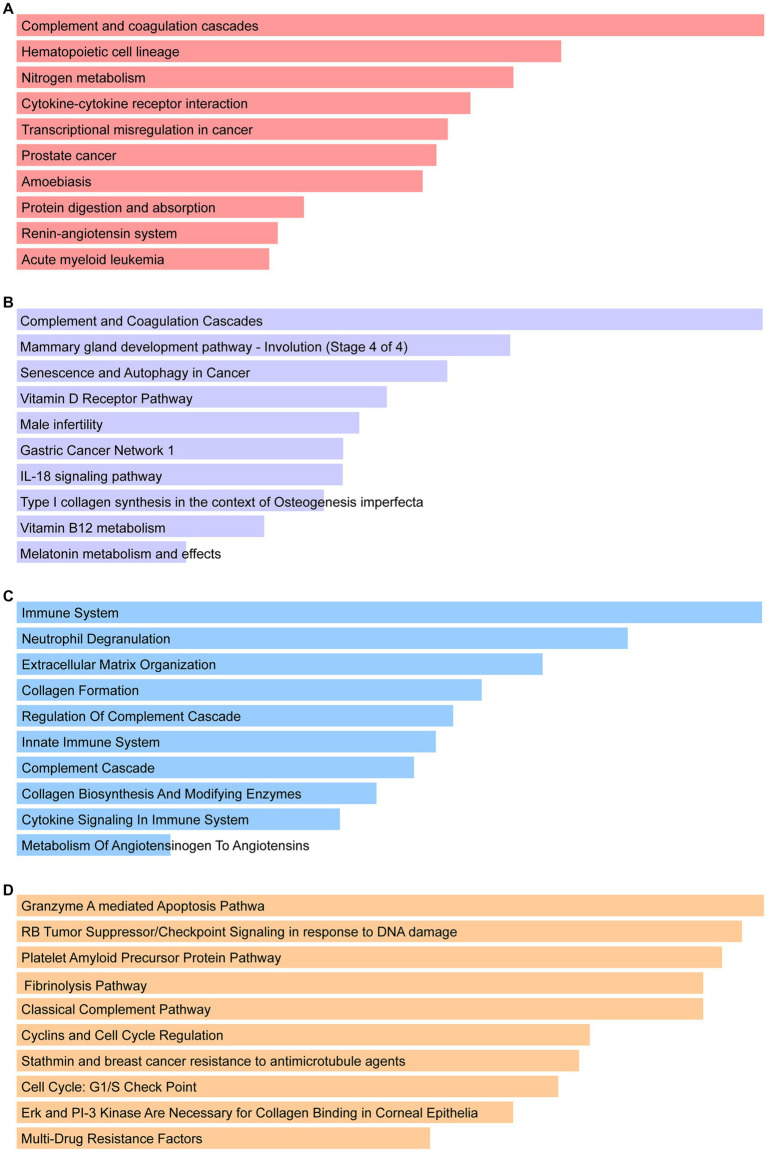
Pathway enrichment analysis: **(A)** KEGG, **(B)** WikiPathways, **(C)** Reactome, and the **(D)** BioCarta.

Enrichr was employed for gene ontology and pathway enrichment analysis to discover the biological significance and enriched pathways of the shared DEGs. The gene ontology enrichment method is widely employed to represent interactions between genes and gene ontology terms. The top ten terms in the categories of biological process, molecular function, and cellular component were gathered in Table 2. Figure 3 presents the total ontological analysis as a linear bar graph for each category. In the biological process, the GO terms are important in relation to neutrophils and interferon-gamma. Pathway analysis demonstrates that the organism responds to its inherent modifications. To assemble the most enriched pathways of the common DEGs, four global databases (KEGG, WikiPathways, Reactome, and BioCarta) were used. Table 3 summarizes the top ten pathways identified by pathway analysis, and the results are presented more intuitively in the bar graph of Figure 4. Acute myeloid leukemia, the immune system, and cyclins and cell cycle regulation pathways are critical in the analysis.

**Table 2:** Ontological analysis of the mutual DEGs between CRC and COVID-19.

Category	Term	*p*-value	Genes
GO Biological Process	Neutrophil degranulation (GO:0043312)	1.82E-09	GSN/TNFAIP6/ANXA3/SLC11A1/HSPA6/MMP9/CEACAM1/PLAU/TCN1/CEACAM6/ANPEP/P2RX1/S100A12/CTSG/S100P/CD14/S100A9/S100A8/ELANE/CD177
	Neutrophil activation involved in immune response (GO:0002283)	2.10E-09	
	Neutrophil mediated immunity (GO:0002446)	2.33E-09	
	Regulation of inflammatory response (GO:0050727)	1.17E-08	MACIR/TNFAIP6/SERPINE1/OSM/MMP9/SBNO2/GPER1/PDCD4/S100A12/ENPP3/S100A9/S100A8/ELANE
	Cytokine-mediated signaling pathway (GO:0019221)	2.55E-06	IFITM3/IL1RN/IFITM1/CCL23/TNFRSF12A/MAOA/IL1R2/IFI6/OSM/MMP9/SOCS3/MT2A/CA1/CEACAM1/COL1A2/OAS3/PDCD4/CTSG
	Regulation of interferon-gamma production (GO:0032649)	5.82E-06	CD2/SLC11A1/SLAMF6/CD14/INHBA/VSIR/LGALS9C
	Negative regulation of viral entry into host cell (GO:0046597)	8.66E-06	IFITM3/IFITM1/GSN/LY6E
	Defense response to bacterium (GO:0042742)	1.24E-05	ANXA3/SLC11A1/OAS3/SERPINE1/S100A12/CTSG/S100A9/S100A8/ELANE
	Negative regulation of viral life cycle (GO:1903901)	1.73E-05	IFITM3/IFITM1/GSN/LY6E
	Regulation of cell migration (GO:0030334)	2.56E-05	ACVRL1/IFITM1/SERPINE1/SEMA3B/MMP9/VSIR/CXCL16/COL1A1/CEACAM1/PLAU/CEACAM6/GPER1/SGK1
GO Cellular Component	Collagen-containing extracellular matrix (GO:0062023)	1.11E-08	COL17A1/C1QB/C1QA/GDF15/SERPINE1/SEMA3B/CLU/MMP9/LOXL1/COL1A1/COL1A2/MMRN1/COL7A1/CTSG/S100A9/S100A8/ELANE
	Secretory granule lumen (GO:0034774)	9.41E-06	GSN/MMRN1/TCN1/SERPINE1/HSPA6/S100A12/CTSG/S100P/CLU/S100A9/S100A8/ELANE
	Specific granule (GO:0042581)	3.08E-04	CEACAM1/PLAU/TCN1/ANXA3/P2RX1/ELANE/CD177
	Cytoplasmic vesicle lumen (GO:0060205)	3.27E-04	GSN/HSPA6/S100A12/S100P/S100A9/S100A8
	Tertiary granule (GO:0070820)	3.58E-04	CEACAM1/TNFAIP6/PLAU/TCN1/SLC11A1/MMP9/CD177
	Secretory granule membrane (GO:0030667)	3.71E-04	CEACAM1/PLAU/CEACAM6/ANPEP/SLC11A1/P2RX1/CA4/CD14/CD177
	Tertiary granule membrane (GO:0070821)	0.0027972	CEACAM1/PLAU/SLC11A1/CD177
	Microtubule (GO:0005874)	0.0034919	TPX2/KIF18B/KIFC1/PLK1/CDK1/KIF20A
	Phagocytic vesicle membrane (GO:0030670)	0.0055745	ANXA3/SLC11A1/TAP1
	Anchored component of plasma membrane (GO:0046658)	0.0059288	CA4/CD14/CD177
GO Molecular Function	RAGE receptor binding (GO:0050786)	4.07E-05	S100A12/S100A9/S100A8
	Serine-type peptidase activity (GO:0008236)	6.65E-05	PLAU/HTRA3/GZMA/PCSK9/CTSG/MMP9/ELANE
	Carbonate dehydratase activity (GO:0004089)	7.91E-05	CA12/CA1/CA4
	Serine-type endopeptidase activity (GO:0004252)	2.00E-04	PLAU/GZMA/PCSK9/CTSG/MMP9/ELANE
	Icosatetraenoic acid binding (GO:0050543)	9.34E-04	S100A9/S100A8
	Arachidonic acid binding (GO:0050544)	9.34E-04	S100A9/S100A8
	Cobalamin binding (GO:0031419)	9.34E-04	TCN2/TCN1
	Endopeptidase activity (GO:0004175)	0.0010049	ADAMTS2/ADAM28/PLAU/HTRA3/GZMA/PCSK9/CTSG/MMP9/ELANE
	Icosanoid binding (GO:0050542)	0.0013009	S100A9/S100A8
	ATP binding (GO:0005524)	0.0018243	ACVRL1/ABCB1/KIFC1/OAS3/PLK1/HSPA6/TAP1/TRIP13

**Table 3:** Pathway enrichment analysis of mutual DEGs.

Category	Term	*p*-value	Genes
KEGG 2021 Human	Complement and coagulation cascades	5.38E-06	C1QB/C1QA/CR2/CFH/PLAU/SERPINE1/CLU
	Hematopoietic cell lineage	1.45E-04	CD2/CR2/ANPEP/IL1R2/CD14/MS4A1
	Nitrogen metabolism	3.15E-04	CA12/CA1/CA4
	Cytokine-cytokine receptor interaction	6.32E-04	ACVRL1/IL1RN/CCL23/TNFRSF12A/GDF15/IL1R2/OSM/INHBA/CXCL16
	Transcriptional misregulation in cancer	9.13E-04	PLAU/IL1R2/ZBTB16/CD14/MMP9/ETV4/ELANE
	Prostate cancer	0.0010972	PLAU/IL1R2/TCF7/E2F1/MMP9
	Amoebiasis	0.0013735	COL1A1/COL1A2/IL1R2/CTSG/CD14
	Protein digestion and absorption	0.0094521	COL17A1/COL1A1/COL1A2/COL7A1
	Renin-angiotensin system	0.0144123	ANPEP/CTSG
	Acute myeloid leukemia	0.0165684	ZBTB16/TCF7/CD14
WikiPathways Human	Complement and coagulation cascades	3.92E-07	C1QB/C1QA/CR2/CFH/PLAU/SERPINE1/CLU
	Mammary gland development pathway - involution (stage 4 of 4)	5.79E-05	SOCS3/E2F1/MMP9
	Senescence and autophagy in cancer	2.00E-04	COL1A1/GSN/PLAU/SERPINE1/E2F1/INHBA
	Vitamin D receptor pathway	6.67E-04	CEACAM1/ABCB1/SEMA3B/G0S2/CD14/S100A9/S100A8
	Male infertility	0.001152	ABCB1/TCN2/EPSTI1/TRIP13/CLU/MMP9
	Gastric cancer network 1	0.0015759	TPX2/UBE2C/S100P
	IL-18 signaling pathway	0.0015911	COL1A1/SOCS3/COL1A2/RGS16/ACACB/MMP9/S1PR4/CXCL16
	Type I collagen synthesis in the context of osteogenesis imperfecta	0.0022987	COL1A1/ADAMTS2/COL1A2
	Vitamin B12 metabolism	0.0074793	TCN2/TCN1/SERPINE1
	Melatonin metabolism and effects	0.0352811	PER1/MAOA
Reactome	Immune system	1.14E-10	IFITM3/C1QB/C1QA/IL1RN/IFITM1/CFH/KLRB1/TNFAIP6/MAOA/IFI6/CLU/SOCS3/MT2A/CA1/PLAU/ANPEP/S100A12/SLAMF6/CTSG/ELANE/CD177/CR2/GSN
	Neutrophil degranulation	7.04E-09	GSN/TNFAIP6/SLC11A1/HSPA6/RNASE1/MMP9/CEACAM1/PLAU/TCN1/CEACAM6/ANPEP/P2RX1/S100A12/CTSG/S100P/S100A9/S100A8/ELANE/CD177
	Extracellular matrix organization	9.60E-08	COL17A1/PCOLCE2/SERPINE1/MMP9/LOXL1/COL1A1/CAPN13/ADAMTS2/CEACAM1/COL1A2/CEACAM6/COL7A1/CTSG/ELANE
	Collagen formation	6.26E-07	COL17A1/COL1A1/ADAMTS2/COL1A2/PCOLCE2/COL7A1/MMP9/LOXL1
	Regulation of complement cascade 977,606	1.50E-06	C1QB/C1QA/CR2/CFH/CLU/ELANE
	Innate immune system	2.56E-06	C1QB/C1QA/CR2/GSN/CFH/TNFAIP6/SLC11A1/HSPA6/CLU/RNASE1/MMP9/CEACAM1/PLAU/TCN1/CEACAM6/ANPEP/P2RX1/S100A12/CTSG/S100P/S100A9/S100A8
	Complement cascade	5.00E-06	C1QB/C1QA/CR2/CFH/CLU/ELANE
	Collagen biosynthesis and modifying enzymes	1.59E-05	COL17A1/COL1A1/ADAMTS2/COL1A2/PCOLCE2/COL7A1
	Cytokine signaling in immune system	4.92E-05	IFITM3/IL1RN/IFITM1/TNFRSF12A/MAOA/IL1R2/IFI6/MMP9/SOCS3/MT2A/CA1/COL1A2/OAS3/PDCD4/S100A12/CTSG/S100A9
	Metabolism of angiotensinogen to angiotensins	0.0089463	ANPEP/CTSG
BioCarta	Granzyme A mediated apoptosis pathway	0.0039824	SET/GZMA
	RB Tumor suppressor/checkpoint signaling in response to DNA damage	0.0046818	E2F1/CDK1
	Platelet amyloid precursor protein pathway	0.0054336	PLAU/SERPINE1
	Fibrinolysis pathway	0.0062368	PLAU/SERPINE1
	Classical complement pathway	0.0062368	C1QB/C1QA
	Cyclins and cell cycle regulation	0.0144123	E2F1/CDK1
	Stathmin and breast cancer resistance to antimicrotubule agents	0.0156408	CD2/CDK1
	Cell cycle: G1/S check point	0.0182269	E2F1/CDK1
	Erk and PI-3 kinase are necessary for collagen binding in corneal epithelia	0.02541	GSN/ZYX
	Multi-drug resistance factors	0.0470557	ABCB1

### 3.3. PPI network analysis and recognition of hub genes

**Figure 5:**
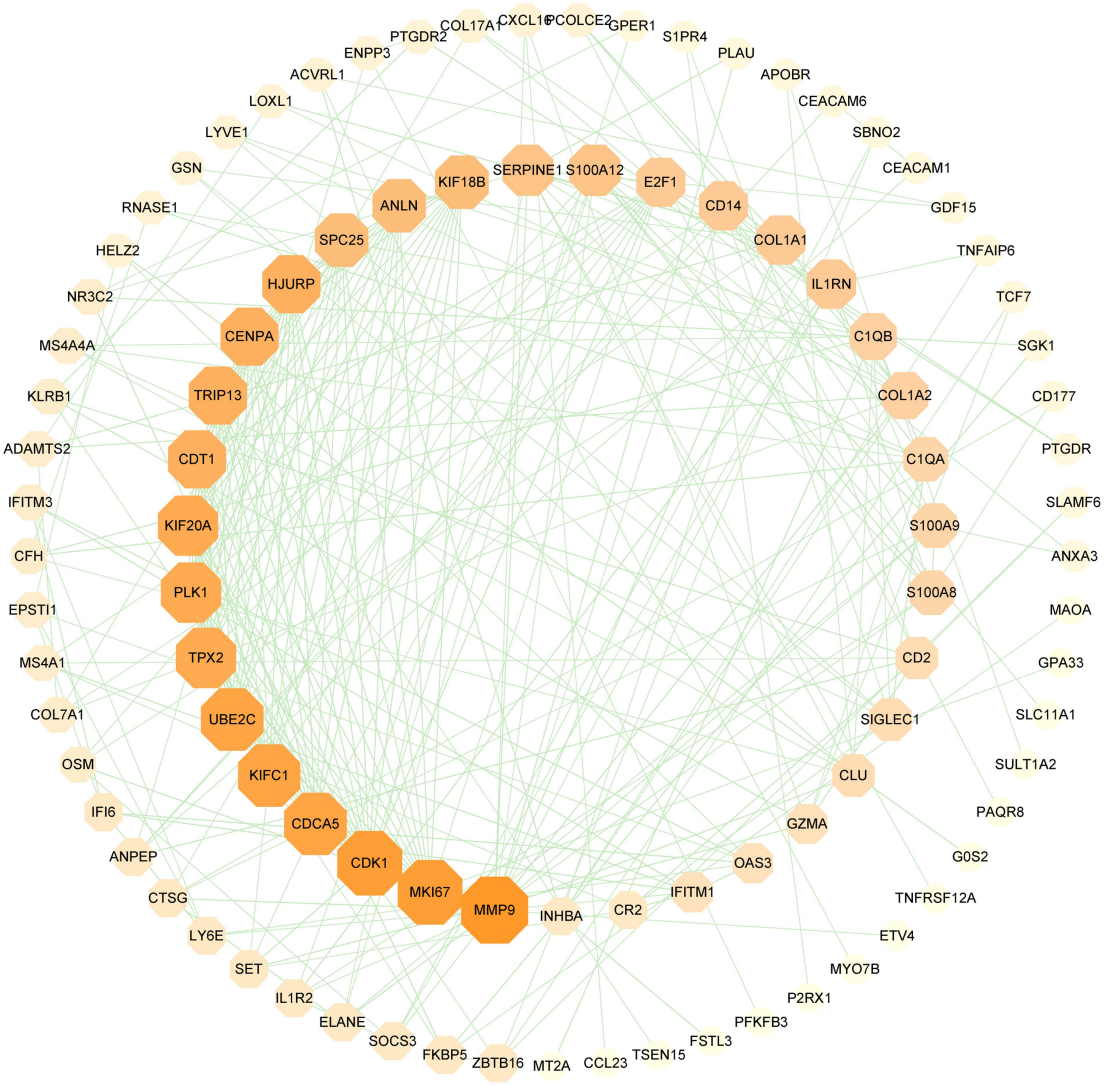
PPI network of SARS-CoV-2 and CRC shared DEGs. The octagons in the figure represent DEGs, while the edges represent node interactions. The PPI network comprises 151 nodes and 286 edges and the size and color depth of the octagon indicate the extent to which proteins are connected.

**Figure 6:**
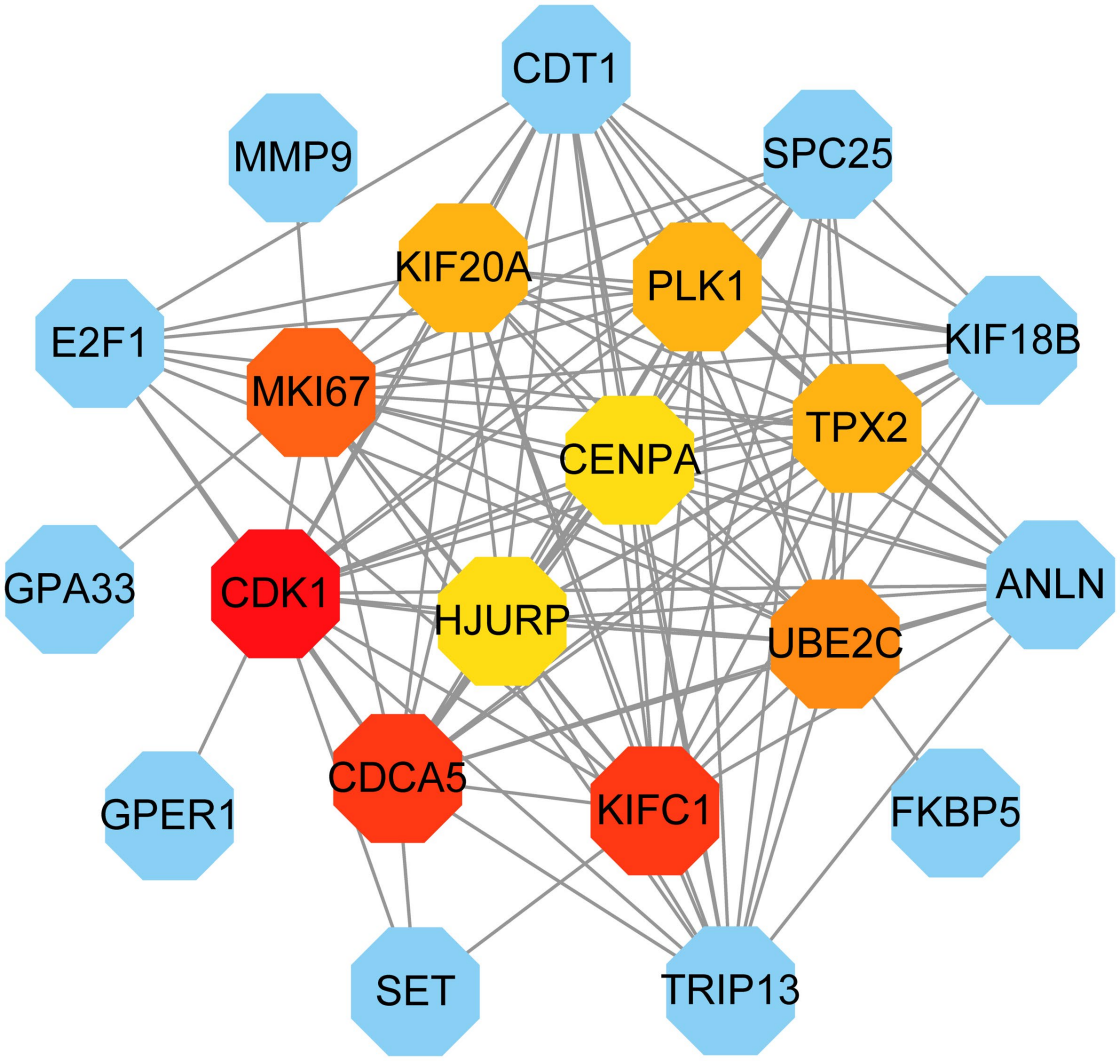
Identification of the hub genes from the PPI network. Here, the highlighted top 10 hub genes and their interactions with other molecules are shown by the red, orange, and yellow nodes (their rank was indicated by a gradient from red to yellow). The network consists of 21 nodes and 117 edges.

PPI network can visually show the correlation between different proteins, indicating the potential mechanism of protein action. Key proteins that affect how cells and systems function biologically have been discovered due to the assessment and analysis of the PPI network (39). The PPI network was created with STRING based on common DEGs and visualized in Cytoscape. The results are shown in Figure 5, which includes 151 nodes and 286 edges. In the PPI network, the majority of interconnected nodes are referred to as hub genes. A submodule network was further constructed with the latest MCC procedure of the Cytohubba plugin in Cytoscape to analyze potential hub genes. The top 10 genes were listed as the most influential genes, including CDK1, KIFC1, CDCA5, MKI67, UBE2C, PLK1, TPX2, KIF20A, HJURP, CENPA (Figure 6).

### 3.4. Determination of transcriptional factors and miRNAs engaged

**Figure 7:**
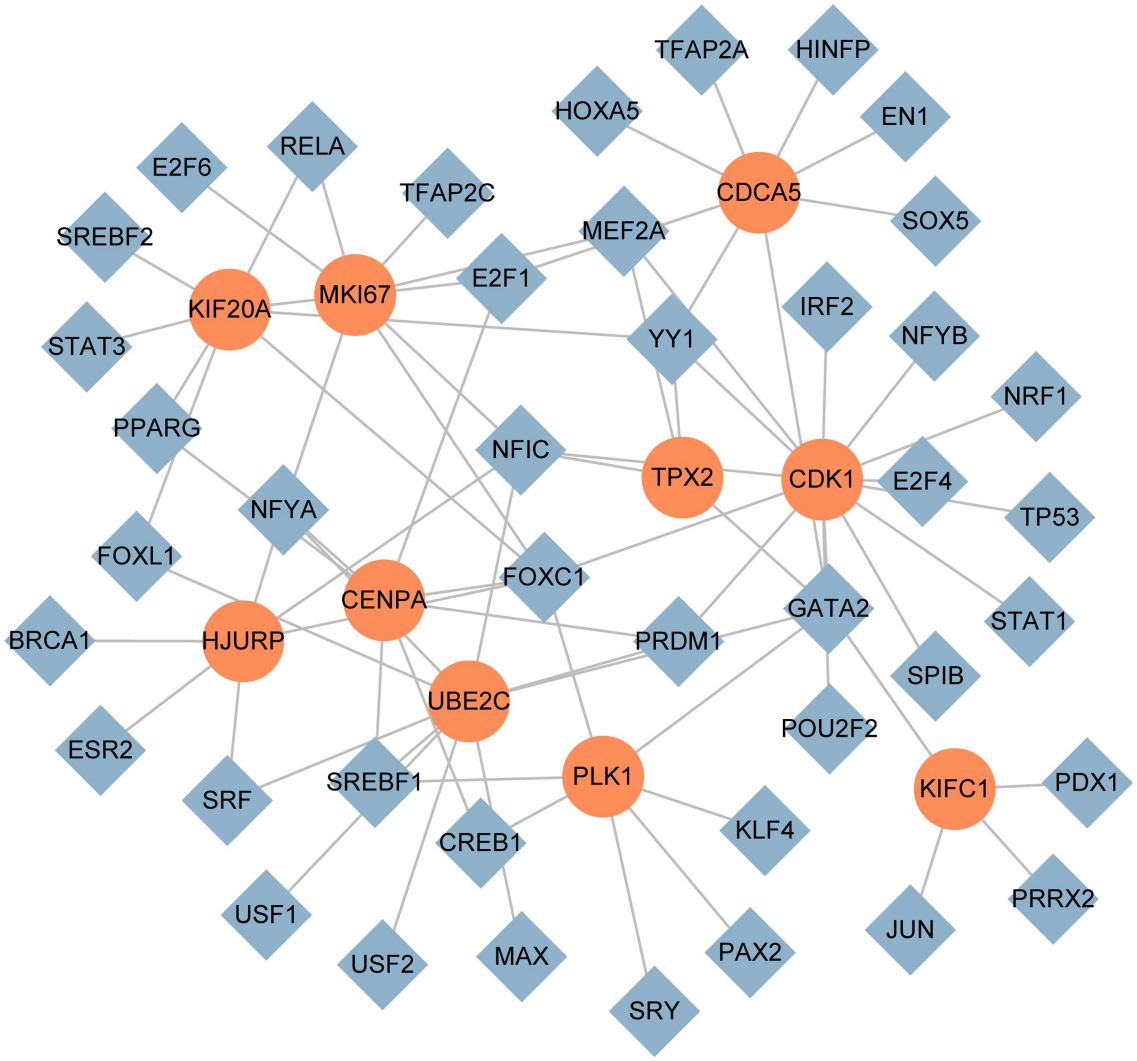
The DEG-TF cohesive regulatory interaction network. The diamond shape nodes in this diagram represent TFs, while the circle nodes represent gene symbols interacting with TFs.

**Figure 8:**
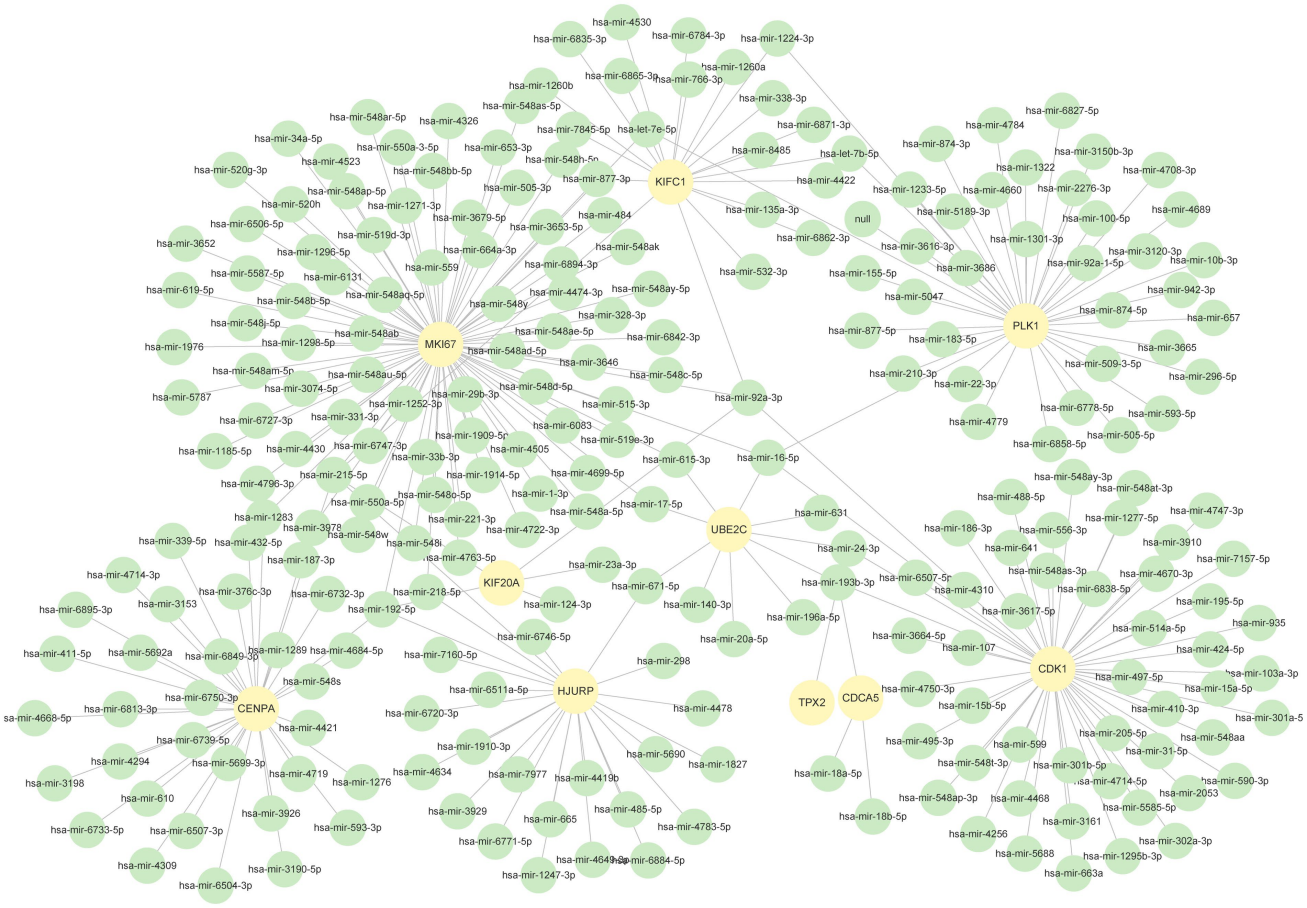
The DEGs-miRNAs regulation interaction network. The green circle nodes in the figure denote miRNAs, whereas the yellow circles represent the genes that interact with miRNAs.

We used NetworkAnalyst to identify regulatory TFs and miRNAs to characterize the significant alterations at the transcriptional level and better comprehend the regulatory key proteins. Figures 7, 8 depict the interactions of TF regulators and miRNA regulators with hub genes. Forty-two TFs and 248 miRNA regulatory signals were projected to affect numerous common differential genes, implying that they interact.

### 3.5. Prediction of potential therapeutic agents and exploration of gene-disease associations

**Figure 9:**
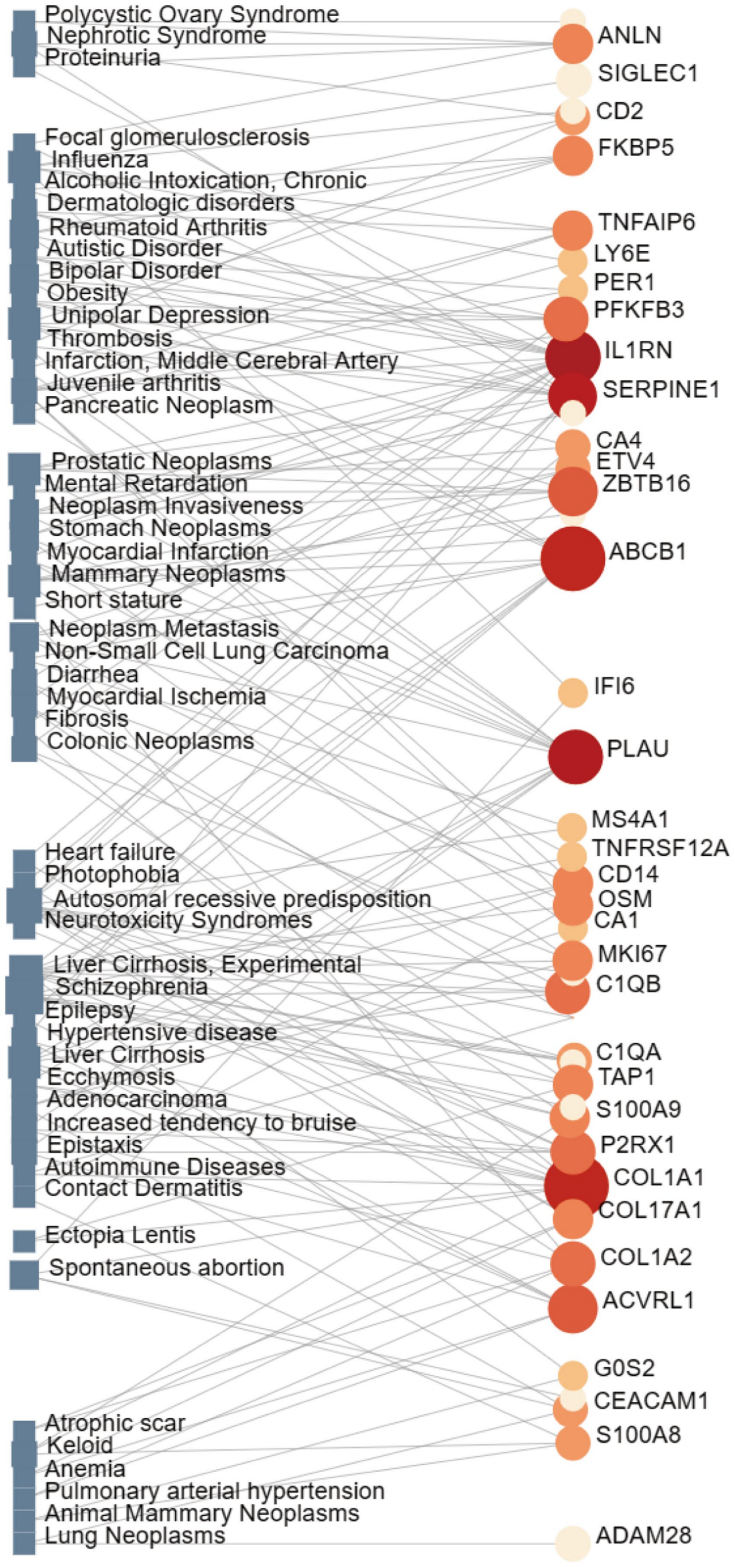
The network of gene-disease associations represents diseases linked by common DEGs. The square node indicates the disease, and the circular node defines the subsequent gene symbols.

Interactions between drugs and proteins play an important role in understanding and recommending structural features for receptor sensitivity. Based on DSigDB, which has the most gene sets connected to drugs/compounds to date, Enrichr was used to screen out the top ten drug molecules with *p*-values from the common DEGs (Table 4). The ten potential drugs include troglitazone, estradiol, progesterone, calcitriol, genistein, dexamethasone, lucanthone, resveratrol, retinoic acid, phorbol 12-myristate 13-acetate. These drugs have the potential to be employed in the treatment of COVID-19 and CRC. Deciphers the connection between genes and disease begins with the development of technologies for illness therapy. DisGeNET is a platform for managing knowledge that unifies and standardizes information on genes and variants linked to disease from many sources. Based on DisGeNET, gene-disease association analysis was performed by NetworkAnalyst. The results showed that lung neoplasms, autoimmune diseases, liver cirrhosis, unipolar depression, and colonic neoplasms were most concordant with the common DEGs (Figure 9).

**Table 4:** The recommended drugs for COVID-19 and CRC.

Name	*p*-value	Chemical formula	Structure
Troglitazone CTD 00002415	1.11E-16	C_24_H_27_NO_5_S	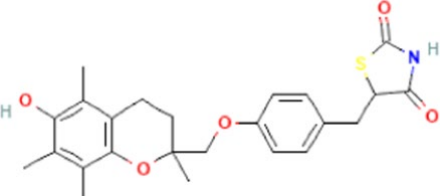
Estradiol CTD 00005920	3.00E-14	C_18_H_24_O_2_	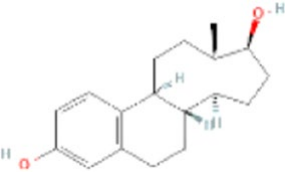
Progesterone CTD 00006624	4.86E-12	C_21_H_30_O_2_	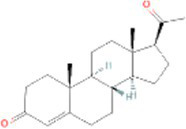
Calcitriol CTD 00005558	1.05E-11	C_27_H_44_O_3_	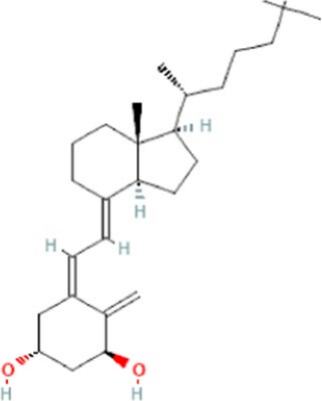
Genistein CTD 00007324	3.57E-11	C_15_H_10_O_5_	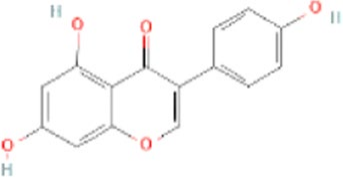
Dexamethasone CTD 00005779	1.24E-09	C_22_H_29_FO_5_	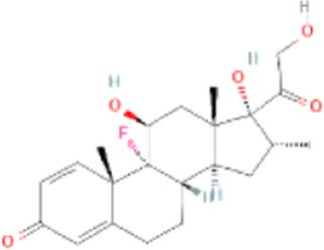
Lucanthone CTD 00006227	1.85E-09	C_20_H_24_N_2_OS	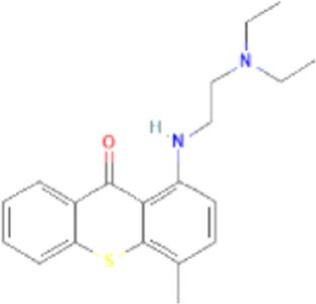
Resveratrol CTD 00002483	2.98E-09	C_14_H_12_O_3_	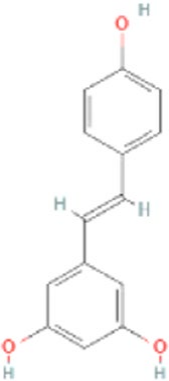
Retinoic acid CTD 00006918	8.41E-09	C_20_H_28_O_2_	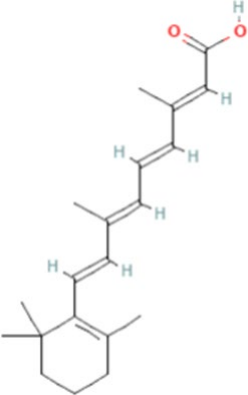
Phorbol 12-myristate 13-acetate CTD 00006852	1.17E-08	C_36_H_56_O_8_	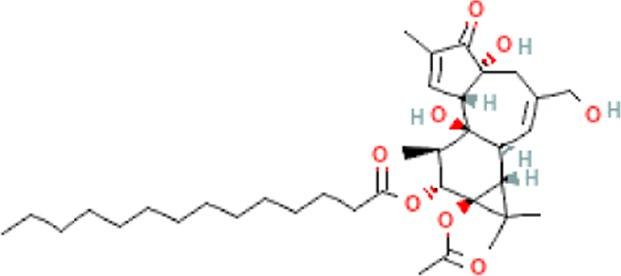

## 4. Discussion

The COVID-19 epidemic, caused by SARS-CoV-2, has been designated a worldwide emergency due to the damage for its quick spread and high mortality (10). CRC is the largest cause of cancer mortality worldwide, and it is one of the cancers whose incidence is increasing, accounting for 11% of all cancer diagnoses (40). Previous research has shown that cancer patients are more sensitive to infection than individuals without cancer, and Chinese patients with colorectal cancer have a significant risk of infection (18, 20, 41). In this study, we investigated the potential interactions between CRC and COVID-19 using bioinformatics and systems biology techniques. We screened 1,668 and 1,674 DEGs from GSE196822 and GSE142279 datasets, respectively, and identified 161 common DEGs. Then, based on common DEGs, we analyzed GO, pathways, PPI network, hub genes, TF-gene interaction network, miRNA-gene coordination node network, candidate drugs, and gene-disease.

GO terms were selected with mutual DEGs and based on *p*-values. For the biological process, neutrophil degranulation, neutrophil activation involved in immune response, neutrophil mediated immunity, and regulation of interferon-gamma production were important GO terms. Neutrophils perform an essential part in the course of COVID-19 infection. The immune response of COVID-19 patients with different severity levels includes increased neutrophil degranulation (42). By releasing neutrophil extracellular traps (NETs), neutrophils recruited to infection sites protect against viral challenges (43). NETs are condensed extracellular chromatin filaments composed of nuclear and cytoplasmic proteins, which are associated with many gastrointestinal diseases, including CRC. It was found that NET formation not only increases the proliferation of CRC cells but also stimulates the transfer process (44). Interferon-gamma, an antiviral agent, is an immunomodulator and plays a key role in humoral and cellular immunity. It has also been shown to be possible to try broad-spectrum antiviral therapy including COVID-19 (45). Under cellular component, collagen-containing extracellular matrix and secretory granule lumen were the top two GO terms. In molecular function, RAGE receptor binding and serine-type peptidase activity were the top two GO terms.

A total of 161 common DEGs were used to search for similar pathways in COVID-19 and CRC. In KEGG pathways, acute myeloid leukemia is significant. Acute myeloid leukemia (AML) is a deadly hematologic cancer. Patients with AML have substantial immunosuppression, and infections are frequently carried on by harsh chemotherapy treatment and disease-related reduced immunity (46). According to the literature, COVID-19 is usually present in a severe clinical form in AML patients, who also frequently have respiratory distress and a very high mortality rate (47). WikiPathways results show that vitamin B12 metabolism and melatonin metabolism and effects are the two pathways of significant importance. In addition to acting as an immunomodulator to regulate cellular immune responses and support hematopoiesis, vitamin B12 can inhibit viral replication in host cells (48, 49). According to Shakeri et al., clinical outcomes in COVID-19 patients are impacted by the host immunological response to viral infections, inflammatory activity, and serum levels of micronutrient deficiencies such as zinc and vitamin B12 deficiency (50). Melatonin is the primary neurohormone secreted by the pineal gland that regulates the sleep–wake cycle. It is also a multifunctional hormone with immunomodulatory, anti-inflammatory, and antiapoptotic properties, as well as having an impact on the metabolism of most organs (51, 52). A randomized clinical trial including 96 COVID-19 patients showed that although melatonin was not associated with a reduction in clinical symptoms and death, melatonin plus standard therapy significantly improved sleep quality and saturation of peripheral oxygen (SpO2) in COVID-19 patients treated for 7 days compared to standard therapy alone. And the effect of melatonin on oxygen delivery and its utilization in tissues may be responsible for the significant improvement in blood oxygen saturation (53). Under Reactome pathways, the immune system and metabolism of angiotensinogen to angiotensins show their importance. When the body is infected with COVID-19, the innate and adaptive immune systems make an effort to eradicate the virus. However, occasionally, the virus interacts with the body in a way that stimulates immune and non-immune cells, resulting in an overactive immunological response (54). Some patients with severe COVID-19 may also experience an overactive cytokine storm or “cytokine release syndrome” caused by an imbalance in the immune system regulation (55). Angiotensinogen, the starting substrate, is cleaved by renin to angiotensin I (Ang I). Ang I is then cleaved by angiotensin-converting enzyme (ACE) to angiotensin II (Ang II), which is metabolized by ACE2 to the beneficial peptide angiotensin 1–7 (Ang 1–7), (56). Importantly, SARS-CoV-2 attacks alveolar cells *via* ACE2 receptors, causing lung infection and leading to COVID-19 (57). In BioCarta pathways, cyclins and cell cycle regulation pathway is critical. COVID-19 patients showed some specific differential features in the expression patterns of some cell cycle-related genes compared to other respiratory diseases. It is important to note that cell cycle characteristics predominated in blood leukocytes (B, T, and natural killer cells) of COVID-19 patients and correlated with their severity and disease trajectory (58).

The PPI network was constructed by using DEGs genes to realize the shared biological functional characteristics of proteins and predict potential drugs. Based on the PPIs network and the MCC method, the top ten hub genes are CDK1, KIFC1, CDCA5, MKI67, UBE2C, PLK1, TPX2, KIF20A, HJURP, and CENPA. Cyclin-dependent kinase 1 (CDK1) is the sole essential required cyclin-dependent kinase that enables the G2-M transition and regulates G1 progression and G1-S transition (59). It has been demonstrated that not only does CDK1 regulate the cell cycle, but it also plays a part in tumor cell proliferation (60–62). Zhang et al. claimed that CDK1 is overexpressed in CRC cells and is apoptosis-sensitive (61). Furthermore, Tong et al. showed that dipeptidyl peptidase 3 (DPP3)/CDK1 contributes to the advancement of CRC by modulating cell proliferation, apoptosis, and migration (62). Kinesin family member C1 (KIFC1) is involved in multiple biological functions and plays a key role in cancer cell centrosome clustering (63). In CRC patients, KIFC1 can regulate ZW10 interacting kinetochore protein (ZWINT) to promote CRC progression and may induce poor prognosis (64). Cell division cycle associated 5 (CDCA5), originally recognized as a substrate that promotes late complexes, has been reported to be associated with the development and progression of multiple human cancers (65). Shen et al. showed that CDCA5 expression was increased in CRC patients and that overexpression of CDCA5 was associated with poor patient survival. Activation of the extracellular signal-regulated kinase (ERK) pathway may show a beneficial role in the oncogenic activity of CDCA5 (66). MKI67, or proliferation marker protein Ki-67, is a nuclear protein that is expressed in all proliferative vertebrate cells and is commonly used as a biomarker to quantify the fraction of proliferating cells to grade tumors (67). MKI67 expression is a late marker of cell cycle entry and has a key role in many CRC-associated genes (68). Downregulation of MKI67 will inhibit cell growth in CRCs (69). The ubiquitin conjugating enzyme E2 C (UBE2C) modifies cellular short-lived or abnormal proteins. Rectal cancer with overexpressed UBE2C regulates miR-381 expression, encouraging rectal carcinoma cell proliferation and invasion (70). Polo-like kinase 1 (PLK1) is a conserved Ser/Thr kinase that has evolved over time and is well known for its function in controlling the cell cycle. It is mostly expressed during the G2/S and M stages of the cell cycle (71). PLK1, which has been found to be overexpressed in numerous cancers, regulates several important transcription factors and promotes cell proliferation, transformation, and epithelial-to-mesenchymal transition (71, 72). In CRC, upregulated PLK1 signaling is frequently accompanied by dysregulation of cell cycle-related pathways. And abnormal PLK1 signaling has been linked to recurrence and a poor prognosis in CRC patients (73). Targeting protein for Xenopus kinesin-like protein 2 (TPX2) is an indirect target of miR-485-3p prediction and has an important part in mitotic spindle assembly and cancer progression. It has been demonstrated that TPX2 is overexpressed in several cancers and highly expressed in CRC tissues (74). The kinesin family member 20A (KIF20A), which is found on chromosome 5q31.2, is crucial for the occurrence and growth of malignancies. KIF20A is overexpressed in CRC tissues and is significantly linked to patients’ poor prognoses. While silencing KIF20A expression can prevent CRC cell lines from migrating and proliferating by activating the janus kinases (JAKs)/signal transducer and activator of transcription 3 (STAT3) signaling pathway (75). The centromeric protein holliday junction recognition protein (HJURP, or hFLEG1) is essential for the incorporation and maintenance of the histone H3-like variant centromere protein A (CENPA) in centromeres. HJURP knockdown reduces CRC cell migration, proliferation, invasion, and tumorigenicity; therefore, it may be used as a prognostic biomarker and a new target for therapeutic development (76). And CENPA can recruit histone acetyltransferase (HAT) general control of amino acid synthesis (GCN)-5 to the karyopherin α_2_ subunit gene (*KPNA2*) promoter region to epigenetically activate its transcriptional activity, resulting in glycolysis and malignant growth in colon cancer (CC) cells (77). Therefore, these hub genes are likely to be potential biomarkers.

To identify the transcriptional and post-transcriptional regulators of the hub genes, we also explore the interactions between the TFs–gene and miRNAs. Among TFs, NFIC, GATA2, YY1, NFYA, E2F1, PRDM1, MEF2A and SREBF1 are associated with cancer (78–85). Nuclear factor I C (NFIC) belongs to the nuclear factor I family. One study reported that NFIC was a low expression in human Lung Squamous Cell Carcinoma (LUSC) tissues and cell lines, and it inhibited the proliferation of LUSC cells and promoted apoptosis *in vitro* and *in vivo*. Additionally, NFIC prevented LUSC cells from migrating and invading other tissues (78). E2F1 is a transcription factor that controls cell cycle progression and was the first member of the E2F transcription factors (E2Fs) to be discovered. It is highly expressed in the majority of cancer cells and prompts the transcription of kinases relevant to the cell cycle. E2F1 regulates the cell cycle in CRC by encouraging spindle construction, where E2F1-induced stathmin1 and transforming acidic coiled-coil-containing protein 3 (TACC3) improve the stability of spindle fiber (82). For DEG-miRNAs, hsa-mir-193b-3p, hsa-mir-192-5p, hsa-mir-215-5p, and hsa-mir-16-5p are associated with cancer. Overexpression of hsa-mir-193b-3p inhibited lung cancer cell invasion and migration and reduced their clonogenic ability. In contrast, hsa-mir-193b-3p inhibition boosted lung cancer’s capacity for metastatic spread and colony formation (86). In cases of colorectal cancer and other malignancies, hsa-mir-192/215-5p can function as a tumor suppressor (87). For hsa-mir-16-5p, in addition to its association with cancer, it was also shown to affect SARS-CoV-2 infection possibly by regulating the ACE2 receptor related network (88, 89). Using data based on single-cell RNA-seq, Li et al. identified hsa-mir-16-5p as one of the probable virus-targeting miRNAs in various cell types in bronchoalveolar lavage fluid samples (89). The previous study also has shown that miRNA hsa-mir-155-5p leads to B cells and T cells activation and exerts immune functions (90). Hsa-mir-34a-5p is one of the promising therapeutics for SARS-CoV-2 (91).

To determine the relationship between significant DEGs and various diseases, we completed a gene-disease analysis. The results showed various types of disease associated with COVID-19, including the brain, blood, skin, heart, liver, joints, and different types of tumors. Brain-related diseases include schizophrenia, unipolar depression, bipolar disorder, epilepsy, and autistic disorder. Individuals with a diagnosis of schizophrenia spectrum disorder were shown to have a higher risk of mortality in a study of adults with SARS-CoV-2 positive test results in a large New York medical system (92). In particular, anxiety disorders are becoming more common than ever before due to the psychosocial impacts of SARS-CoV-2, which in turn may precipitate the onset of a number of co-morbid psychiatric conditions like depression and bipolar disorder (93). In this sense, viral infections are linked to a higher incidence of anxiety and/or depression (94). We also discovered skin problems including contact dermatitis. According to solid data, the COVID-19 pandemic has led to an increase in the prevalence of allergic and irritating contact dermatitis. The most typically reported symptoms are skin dryness, itchiness, and redness (95). Furthermore, Grivas et al. used detailed information from nearly 5,000 patients with COVID-19 and cancer to confirm that COVID-19 severity and mortality are high in cancer patients. This was particularly the case for older age, male sex, non-Hispanic non-white racial/ethnic group, worse eastern cooperative oncology group performance status, hematologic malignancies, and selected laboratory measures (96).

Through the common DEGs, we predicted ten drugs, including troglitazone, estradiol, progesterone, calcitriol, genistein, dexamethasone, lucanthone, resveratrol, retinoic acid, phorbol 12-myristate 13-acetate. Several substances have been evaluated as CRC and COVID-19 therapies. Endogenous reproductive hormones estradiol and progesterone are produced in large amounts in the periphery by the adrenal glands and ovaries, and *de novo* by the brain. They have a significant physiological impact by controlling inflammatory behaviors and processes (97). In the azoxymethane/dextran sulfate sodium (AOM/DSS) mouse model, estradiol inhibits the development of CRC and downregulated inflammation (98). And progesterone, which could inhibit CRC proliferation by blocking the cell cycle and inducing apoptosis in turn limits malignant tumor progression (99). In COVID-19, women with high estrogen levels have a lower risk of severe symptoms and even lower mortality (100). Calcitriol, the biologically active form of vitamin D, is effective as a treatment for patients with hypocalcemia or secondary hyperparathyroidism caused by renal insufficiency (101). For other roles, calcitriol can inhibit glycolysis and cell growth in human CRC cells, suggesting an inhibitory role in CRC progression (102). And Elamir et al. have demonstrated improved oxygenation in hospitalized COVID-19 patients treated with calcitriol (103). Genistein is one of the main bioactive substances of soybean isoflavones that exert pharmacological functions (104). It can block cancer cell proliferation by various mechanisms, such as up-regulation of p21 levels and inhibition of tyrosine-specific kinase activity (105, 106). In human CRC cells, genistein exerts anti-invasive and anti-proliferative effects by inhibiting cell proliferation and inducing apoptosis (107). Additionally, RNA transcripts and protein synthesis from viruses like rotavirus can be inhibited by genistein (108). Therefore, genistein has the potential to be used as a lead chemical in the evaluation of new anti-CRC and antiviral drug candidates. Dexamethasone is a drug used to treat a variety of inflammatory diseases by effectively inhibiting the release of substances that cause inflammation, and it has been already in clinical use for cancer prevention (109). In addition, for patients who are receiving mechanical ventilation, the corticosteroid dexamethasone can lower the rate of COVID-19-related mortality in the critical care unit by 35%. Despite the varying efficiency of other combination medications in treating COVID-19 associated with ARDS, dexamethasone is widely employed in several COVID-19 therapy regimens (110). Resveratrol as a traditional Chinese medicine chemical monomer can effectively inhibit colon cancer cell growth by the ROS-dependent ferroptosis pathway (111). Retinoic acid is further processed from vitamin A (retinol) by several enzymes and exists in several stereoisomeric forms. In addition to its role in biological processes such as reproduction, differentiation, embryogenesis, and eye development, retinoic acid signaling has been shown to be a possible therapeutic target for CRC (112). Furthermore, McCreary and Sarohan et al. have also shown that resveratrol and retinoic acid may also be new perspectives for the treatment of COVID-19 (113, 114).

This study explored the correlation between CRC and COVID-19 using bioinformatics and systems biology techniques. Through high-throughput sequencing, this study identified COVID-19 and CRC-related biomarkers and potential drugs, which provide new clues for the treatment of both diseases. It is worth noting that there are still some limitations in this study. First, it is impossible to fully recapitulate the underlying genetic linkages using a computational biology technique because of information and method bias. Second, we currently do not have experiments *in vivo* or *in vitro* to validate the findings, and specific experiments will be conducted in the next step. Last, there is still a long way to go to translate experimental results into clinical applications.

## 5. Conclusion

In this study, the transcriptome datasets of CRC and COVID-19 were used to identify DEGs and elucidated the intrinsic link between the two diseases. A total of 161 common DEGs were found, based on which a PPI network was constructed to recognize the top ten hub genes. These target genes may be biomarkers of CRC or SARS-CoV-2 infection and thus translate into potential drug targets. We also investigated the relationship of these target genes with TFs and miRNAs to better understand their role in disease occurrence and progression. Meanwhile, multiple drug molecules and drug-target interactions of common DEGs were retrieved using the DSigDB database. However, further experiments are needed to verify whether these potential gene targets and drugs can be further used in clinical practice.

## Data availability statement

The datasets presented in this study can be found in online repositories. The names of the repository/repositories and accession number(s) can be found in the article/supplementary material.

## Author contributions

YS, TH, and HP contributed to the conception and design of the study. YS organized the database. TH performed the formal analysis. HP was responsible for the article methodology. AD, TW, JL, XZ, YL, and SX wrote sections of the manuscript. KY conducted the overall supervision and validation. All authors contributed to the article and approved the submitted version.

## Funding

This work was supported by grants from the National multidisciplinary collaborative diagnosis and treatment capacity building project for major diseases (TJZ202104), the Natural Science Foundation of China (82173248, 82272685, 82002967 and 82202260), the Postdoctoral Science the fellowship of China National Postdoctoral Program for Innative Talents (BX20200225), the Project funded by China Postdoctoral Science Foundation (2022TQ0221), the Science and Technology Major Program of Sichuan Province (2022ZDZX0019), the Sichuan Science and Technology Program (2023YFS0128, 2023NSFSC1874, 2021YJ0420), 1.3.5 project for disciplines of excellence, West China Hospital, Sichuan University (ZYJC18008, ZYGD22006), the Sichuan University postdoctoral interdisciplinary Innovation Fund (10822041A2103).

## Conflict of interest

The authors declare that the research was conducted in the absence of any commercial or financial relationships that could be construed as a potential conflict of interest.

## Publisher’s note

All claims expressed in this article are solely those of the authors and do not necessarily represent those of their affiliated organizations, or those of the publisher, the editors and the reviewers. Any product that may be evaluated in this article, or claim that may be made by its manufacturer, is not guaranteed or endorsed by the publisher.

## Footnotes

^1^https://www.ncbi.nlm.nih.gov/

^2^http://jvenn.toulouse.inra.fr/app/index.html

^3^https://maayanlab.cloud/enrichr/

^4^www.string-db.org

^5^http://www.disgenet.org/

## References

[1] Ciotti M Ciccozzi M Terrinoni A Jiang WC Wang CB Bernardini S. The Covid-19 pandemic. Crit Rev Clin Lab Sci. (2020) 57:365–88. doi: 10.1080/10408363.2020.1783198 32645276

[2] Andersen KG Rambaut A Lipkin WI Holmes EC Garry RF. The proximal origin of Sars-Cov-2. Nat Med. (2020) 26:450–2. doi: 10.1038/s41591-020-0820-9, PMID: 32284615 PMC7095063

[3] Li K Meyerholz DK Bartlett JA McCray PB Jr. The Tmprss 2 inhibitor Nafamostat reduces Sars-Cov-2 pulmonary infection in mouse models of Covid-19. MBio. (2021) 12:e00970–21. doi: 10.1128/mBio.00970-21 34340553 PMC8406266

[4] Allam L Ghrifi F Mohammed H El Hafidi N El Jaoudi R El Harti J. Targeting the Grp 78-Dependant Sars-Cov-2 cell entry by peptides and small molecules. Bioinform Biol Insights. (2020) 14:117793222096550. doi: 10.1177/1177932220965505 PMC7585878 33149560

[5] Strollo R Pozzilli P. Dpp4 inhibition: preventing Sars-Cov-2 infection and/or progression of Covid-19? Diabetes Metab Res Rev. (2020) 36:e3330. doi: 10.1002/dmrr.3330, PMID: 32336007 PMC7267128

[6] Behl T Kaur I Aleya L Sehgal A Singh S Sharma N. Cd147-spike protein interaction in Covid-19: get the ball rolling with a novel receptor and therapeutic target. Sci Total Environ. (2022) 808:152072. doi: 10.1016/j.scitotenv.2021.152072, PMID: 34863742 PMC8634688

[7] Wang S Qiu Z Hou Y Deng X Xu W Zheng T. Axl is a candidate receptor for Sars-Cov-2 that promotes infection of pulmonary and bronchial epithelial cells. Cell Res. (2021) 31:126–40. doi: 10.1038/s41422-020-00460-y, PMID: 33420426 PMC7791157

[8] Ou X Liu Y Lei X Li P Mi D Ren L. Characterization of spike glycoprotein of Sars-Cov-2 on virus entry and its immune cross-reactivity with Sars-Cov. Nat Commun. (2020) 11:1620. doi: 10.1038/s41467-020-15562-9, PMID: 32221306 PMC7100515

[9] Cao X. Covid-19: immunopathology and its implications for therapy. Nat Rev Immunol. (2020) 20:269–70. Epub 2020/04/11. doi: 10.1038/s41577-020-0308-3, PMID: 32273594 PMC7143200

[10] Yang L Liu S Liu J Zhang Z Wan X Huang B. Covid-19: Immunopathogenesis and Immunotherapeutics. Signal Transduct Target Ther. (2020) 5:1–8. doi: 10.1038/s41392-020-00243-2 32712629 PMC7381863

[11] Wang D Hu B Hu C Zhu F Liu X Zhang J. Clinical characteristics of 138 hospitalized patients with 2019 novel coronavirus–infected pneumonia in Wuhan, China. JAMA. (2020) 323:1061–9. doi: 10.1001/jama.2020.1585, PMID: 32031570 PMC7042881

[12] Abd El-Raheem GOH Elamin HES Ahmad ZMO Noma M. Spatial–temporal trends of COVID-19 infection and mortality in Sudan. Sci Rep. (2022) 12:16822. doi: 10.1038/s41598-022-21137-z, PMID: 36207365 PMC9540272

[13] Madabhavi I Sarkar M Kadakol N. Covid-19: a review. Monaldi Arch Chest Dis. (2020) 90:248–258. doi: 10.4081/monaldi.2020.1298 32498503

[14] Ma C Dong W Shen B Zhang H. Editorial: Covid-19 and the digestive system. Front Med (Lausanne). (2022) 9:875063. doi: 10.3389/fmed.2022.875063, PMID: 35433728 PMC9006941

[15] Al-Quteimat OM Amer AM. The impact of the Covid-19 pandemic on cancer patients. Am J Clin Oncol. (2020) 43:452–5. doi: 10.1097/COC.0000000000000712, PMID: 32304435 PMC7188063

[16] Naimi A Yashmi I Jebeleh R Imani Mofrad M Azimian Abhar S Jannesar Y. Comorbidities and mortality rate in Covid-19 patients with hematological malignancies: a systematic review and Meta-analysis. J Clin Lab Anal. (2022) 36:e24387. doi: 10.1002/jcla.24387, PMID: 35385130 PMC9102765

[17] Liang W Guan W Chen R Wang W Li J Xu K. Cancer patients in Sars-Cov-2 infection: a Nationwide analysis in China. Lancet Oncol. (2020) 21:335–7. doi: 10.1016/S1470-2045(20)30096-6, PMID: 32066541 PMC7159000

[18] Gao Y Liu M Shi S Chen Y Sun Y Chen J. Cancer is associated with the severity and mortality of patients with Covid-19: a systematic review and meta-analysis. MedRxiv. (2020). doi: 10.1101/2020.05.01.20087031

[19] Xie YH Chen YX Fang JY. Comprehensive review of targeted therapy for colorectal Cancer. Signal Transduct Target Ther. (2020) 5:22. doi: 10.1038/s41392-020-0116-z, PMID: 32296018 PMC7082344

[20] Antikchi MH Neamatzadeh H Ghelmani Y Jafari-Nedooshan J Dastgheib SA Kargar S. The risk and prevalence of Covid-19 infection in colorectal Cancer patients: a systematic review and Meta-analysis. J Gastrointest Cancer. (2021) 52:73–9. doi: 10.1007/s12029-020-00528-3, PMID: 32997314 PMC7524641

[21] Burgueño JF Reich A Hazime H Quintero MA Fernandez I Fritsch J. Expression of Sars-Cov-2 entry molecules Ace2 and Tmprss2 in the gut of patients with Ibd. Inflamm Bowel Dis. (2020) 26:797–808. doi: 10.1093/ibd/izaa085, PMID: 32333601 PMC7188157

[22] Parise R Li YE Nadar RM Ramesh S Ren J Govindarajulu MY. Health influence of Sars-Cov-2 (Covid-19) on Cancer: a review. Acta Biochim Biophys Sin. (2022) 54:1395–405. doi: 10.3724/abbs.2022147, PMID: 36269132 PMC9828497

[23] Barrett T Wilhite SE Ledoux P Evangelista C Kim IF Tomashevsky M. Ncbi geo: archive for functional genomics data sets—update. Nucleic Acids Res. (2012) 41:D991–5. doi: 10.1093/nar/gks1193, PMID: 23193258 PMC3531084

[24] Blanco-Melo D Nilsson-Payant BE Liu W-C Uhl S Hoagland D Møller R. Imbalanced host response to Sars-Cov-2 drives development of Covid-19. Cells. (2020) 181:1036–1045.e9. doi: 10.1016/j.cell.2020.04.026, PMID: 32416070 PMC7227586

[25] Fang H Sun Z Chen Z Chen A Sun D Kong Y. Bioinformatics and systems-biology analysis to determine the effects of coronavirus disease 2019 on Patients with Allergic Asthma. Front Immunol. (2022) 13:5437. doi: 10.3389/fimmu.2022.988479 PMC9537444 36211429

[26] Love MI Huber W Anders S. Moderated estimation of fold change and dispersion for Rna-Seq data with Deseq2. Genome Biol. (2014) 15:1–21. doi: 10.1186/s13059-014-0550-8 PMC4302049 25516281

[27] Smyth GK. “limma: Linear Models for Microarray Data,” in Bioinformatics and Computational Biology Solutions Using R and Bioconductor. eds. Gentleman R. Carey V. J. Huber W. Irizarry R. A. Hedges S. (New York, NY: Statistics for Biology and Health, Springer).

[28] Bardou P Mariette J Escudié F Djemiel C Klopp C. Jvenn: an interactive venn diagram viewer. BMC Bioinformatics. (2014) 15:1–7. doi: 10.1186/1471-2105-15-293 25176396 PMC4261873

[29] Peng J Wang H Lu J Hui W Wang Y Shang X. Identifying term relations cross different gene ontology categories. BMC Bioinformatics. (2017) 18:67–74. doi: 10.1186/s12859-017-1959-3 29297309 PMC5751813

[30] Kuleshov MV Jones MR Rouillard AD Fernandez NF Duan Q Wang Z. Enrichr: a comprehensive gene set enrichment analysis web server 2016 update. Nucleic Acids Res. (2016) 44:W90–7. doi: 10.1093/nar/gkw377, PMID: 27141961 PMC4987924

[31] De Las RJ Fontanillo C. Protein–protein interactions essentials: key concepts to building and analyzing Interactome networks. PLoS Comput Biol. (2010) 6:e1000807. doi: 10.1371/journal.pcbi.1000807 20589078 PMC2891586

[32] Chin C-H Chen S-H Wu H-H Ho C-W Ko M-T Lin C-Y. Cytohubba: identifying hub objects and sub-networks from complex Interactome. BMC Syst Biol. (2014) 8:1–7. doi: 10.1186/1752-0509-8-S4-S11 25521941 PMC4290687

[33] Lambert SA Jolma A Campitelli LF Das PK Yin Y Albu M. The human transcription factors. Cells. (2018) 172:650–65. doi: 10.1016/j.cell.2018.01.029 PMC12908702 29425488

[34] Zhou G Soufan O Ewald J Hancock RE Basu N Xia J. Networkanalyst 3.0: a visual analytics platform for comprehensive gene expression profiling and Meta-analysis. Nucleic Acids Res. (2019) 47:W234–41. doi: 10.1093/nar/gkz240, PMID: 30931480 PMC6602507

[35] Li X-y Wang J-b An H-b Wen M-z You J-x Yang X-t. Effect of Sars-Cov-2 infection on asthma patients. Front Med. (2022) 9:9. doi: 10.3389/fmed.2022.928637 PMC9378965 35983093

[36] Sethupathy P Corda B Hatzigeorgiou AG. Tarbase: a comprehensive database of experimentally supported animal Microrna targets. RNA. (2006) 12:192–7. doi: 10.1261/rna.2239606, PMID: 16373484 PMC1370898

[37] Yoo M Shin J Kim J Ryall KA Lee K Lee S. Dsigdb: drug signatures database for gene set analysis. Bioinformatics. (2015) 31:3069–71. doi: 10.1093/bioinformatics/btv313, PMID: 25990557 PMC4668778

[38] Salnikova LE Chernyshova EV Anastasevich LA Larin SS. Gene-and disease-based expansion of the knowledge on inborn errors of immunity. Front Immunol. (2019) 10:2475. doi: 10.3389/fimmu.2019.02475, PMID: 31695696 PMC6816315

[39] Lu L Liu LP Gui R Dong H Su YR Zhou XH. Discovering common Pathogenetic processes between Covid-19 and Sepsis by bioinformatics and system biology approach. Front Immunol. (2022) 13:975848. Epub 2022/09/20. doi: 10.3389/fimmu.2022.975848, PMID: 36119022 PMC9471316

[40] Sawicki T Ruszkowska M Danielewicz A Niedźwiedzka E Arłukowicz T Przybyłowicz KE. A review of colorectal Cancer in terms of epidemiology, risk factors, development, symptoms and diagnosis. Cancers. (2021) 13:2025. doi: 10.3390/cancers13092025, PMID: 33922197 PMC8122718

[41] Shen Q Wang J Zhao L. To investigate the internal association between Sars-Cov-2 infections and cancer through bioinformatics. Math Biosci Eng. (2022) 19:11172–94. doi: 10.3934/mbe.2022521, PMID: 36124586

[42] Bibert S Guex N Lourenco J Brahier T Papadimitriou-Olivgeris M Damonti L. Transcriptomic signature differences between Sars-Cov-2 and influenza virus infected patients. Front Immunol. (2021) 12:666163. doi: 10.3389/fimmu.2021.666163, PMID: 34135895 PMC8202013

[43] Jenne CN Wong CH Zemp FJ McDonald B Rahman MM Forsyth PA. Neutrophils recruited to sites of infection protect from virus challenge by releasing neutrophil extracellular traps. Cell Host Microbe. (2013) 13:169–80. doi: 10.1016/j.chom.2013.01.005, PMID: 23414757

[44] Khan U Chowdhury S Billah MM Islam KMD Thorlacius H Rahman M. Neutrophil extracellular traps in colorectal Cancer progression and metastasis. Int J Mol Sci. (2021) 22:7260. doi: 10.3390/ijms22147260, PMID: 34298878 PMC8307027

[45] Ozcelik F Tanoglu A Çıracı MZ Ozcelik IK. Use of immune modulator interferon-gamma to support combating Covid-19 pandemic. Int J Coronaviruses. (2020) 1:1–15. doi: 10.14302/issn.2692-1537.ijcv-20-3345

[46] Ferrara F Schiffer CA. Acute Myeloid Leukaemia in Adults. Lancet. (2013) 381:484–95. doi: 10.1016/S0140-6736(12)61727-9 23399072

[47] Marchesi F Salmanton-García J Emarah Z Piukovics K Nucci M López-García A. Covid-19 in adult acute myeloid leukemia patients: a long-term Followup study from the European Hematology Association survey (Epicovideha). Haematologica. (2023) 108:22–33. doi: 10.3324/haematol.2022.280847 35545919 PMC9827164

[48] Maggini S Pierre A Calder PC. Immune function and micronutrient requirements change over the life course. Nutrients. (2018) 10:1531. doi: 10.3390/nu10101531, PMID: 30336639 PMC6212925

[49] Herrmann W Obeid R. Causes and early diagnosis of vitamin B12 deficiency. Dtsch Arztebl Int. (2008) 105:680–5. doi: 10.3238/arztebl.2008.0680, PMID: 19623286 PMC2696961

[50] Shakeri H Azimian A Ghasemzadeh-Moghaddam H Safdari M Haresabadi M Daneshmand T. Evaluation of the relationship between serum levels of zinc, vitamin B12, vitamin D, and clinical outcomes in patients with Covid-19. J Med Virol. (2022) 94:141–6. doi: 10.1002/jmv.27277, PMID: 34406674 PMC8426973

[51] Auld F Maschauer EL Morrison I Skene DJ Riha RL. Evidence for the efficacy of melatonin in the treatment of primary adult sleep disorders. Sleep Med Rev. (2017) 34:10–22. doi: 10.1016/j.smrv.2016.06.005, PMID: 28648359

[52] Zhang R Wang X Ni L Di X Ma B Niu S. Covid-19: melatonin as a potential adjuvant treatment. Life Sci. (2020) 250:117583. doi: 10.1016/j.lfs.2020.117583, PMID: 32217117 PMC7102583

[53] Mousavi SA Heydari K Mehravaran H Saeedi M Alizadeh-Navaei R Hedayatizadeh-Omran A. Melatonin effects on sleep quality and outcomes of Covid-19 patients: An open-label, randomized, controlled trial. J Med Virol. (2022) 94:263–71. doi: 10.1002/jmv.27312, PMID: 34460132 PMC8662261

[54] Xu Z Shi L Wang Y Zhang J Huang L Zhang C. Pathological findings of Covid-19 associated with acute respiratory distress syndrome. Lancet Respir Med. (2020) 8:420–2. doi: 10.1016/S2213-2600(20)30076-X, PMID: 32085846 PMC7164771

[55] Eisen HN Chakraborty AK. Evolving concepts of specificity in immune reactions. Proc Natl Acad Sci. (2010) 107:22373–80. doi: 10.1073/pnas.1012051108, PMID: 21173256 PMC3012479

[56] Patel VB Zhong J-C Grant MB Oudit GY. Role of the Ace2/angiotensin 1–7 Axis of the renin–angiotensin system in heart failure. Circ Res. (2016) 118:1313–26. doi: 10.1161/CIRCRESAHA.116.307708, PMID: 27081112 PMC4939482

[57] Su W-L Lu K-C Chan C-Y Chao Y-C. Covid-19 and the lungs: a review. J Infect Public Health. (2021) 14:1708–14. doi: 10.1016/j.jiph.2021.09.024, PMID: 34700289 PMC8486577

[58] Prado CAS Fonseca DLM Singh Y Filgueiras IS Baiocchi GC Placa DR. Title: integrative systems immunology uncovers molecular networks of the cell cycle that stratify Covid-19 severity. J Med Virol. (2023) 95:e28450. doi: 10.1002/jmv.28450, PMID: 36597912 PMC10107240

[59] Enserink JM Kolodner RD. An overview of Cdk1-controlled targets and processes. Cell Div. (2010) 5:11–41. doi: 10.1186/1747-1028-5-11 20465793 PMC2876151

[60] Yang W Cho H Shin H-Y Chung J-Y Kang ES Lee E-j. Accumulation of cytoplasmic Cdk1 is associated with Cancer growth and survival rate in epithelial ovarian Cancer. Oncotarget. (2016) 7:49481–97. doi: 10.18632/oncotarget.10373 27385216 PMC5226523

[61] Zhang P Kawakami H Liu W Zeng X Strebhardt K Tao K. Targeting Cdk1 and Mek/Erk overcomes apoptotic resistance in Braf-mutant human colorectal Cancerdual targeting Cdk1 and Mek in Braf-mutant colorectal Cancer. Mol Cancer Res. (2018) 16:378–89. doi: 10.1158/1541-7786.MCR-17-0404, PMID: 29233910

[62] Tong Y Huang Y Zhang Y Zeng X Yan M Xia Z. Dpp3/Cdk1 contributes to the progression of colorectal Cancer through regulating cell proliferation, cell apoptosis, and cell migration. Cell Death Dis. (2021) 12:529. doi: 10.1038/s41419-021-03796-4, PMID: 34023852 PMC8141054

[63] Han J Wang F Lan Y Wang J Nie C Liang Y. Kifc1 regulated by Mir-532-3p promotes epithelial-to-Mesenchymal transition and metastasis of hepatocellular carcinoma Via Gankyrin/Akt signaling. Oncogene. (2019) 38:406–20. doi: 10.1038/s41388-018-0440-8, PMID: 30115976 PMC6336682

[64] Akabane S Oue N Sekino Y Asai R Thang PQ Taniyama D. Kifc1 regulates Zwint to promote tumor progression and spheroid formation in colorectal Cancer. Pathol Int. (2021) 71:441–52. doi: 10.1111/pin.13098, PMID: 33819373

[65] Zhang N Pati D. Sororin is a master regulator of sister chromatid cohesion and separation. Cell Cycle. (2012) 11:2073–83. doi: 10.4161/cc.20241, PMID: 22580470 PMC3368859

[66] Shen A Liu L Chen H Qi F Huang Y Lin J. Cell division cycle associated 5 promotes colorectal Cancer progression by activating the Erk signaling pathway. Oncogenesis. (2019) 8:19. doi: 10.1038/s41389-019-0123-5, PMID: 30808873 PMC6391450

[67] Cidado J Wong HY Rosen DM Cimino-Mathews A Garay JP Fessler AG. Ki-67 is required for maintenance of Cancer stem cells but not cell proliferation. Oncotarget. (2016) 7:6281–93. doi: 10.18632/oncotarget.7057, PMID: 26823390 PMC4868756

[68] Sobecki M Mrouj K Colinge J Gerbe F Jay P Krasinska L. Cell-cycle regulation accounts for variability in Ki-67 expression Levelski-67 expression and the cell cycle. Cancer Res. (2017) 77:2722–34. doi: 10.1158/0008-5472.CAN-16-0707, PMID: 28283655

[69] Zeng Q Lei F Chang Y Gao Z Wang Y Gao Q. An oncogenic gene, Snrpa1, regulates Pik3r1, Vegfc, Mki67, Cdk1 and other genes in colorectal Cancer. Biomed Pharmacother. (2019) 117:109076. doi: 10.1016/j.biopha.2019.109076, PMID: 31203132

[70] Niu L Gao C Li Y. Identification of potential Core genes in colorectal carcinoma and key genes in colorectal Cancer liver metastasis using bioinformatics analysis. Sci Rep. (2021) 11:23938. doi: 10.1038/s41598-021-03395-5, PMID: 34907282 PMC8671463

[71] Iliaki S Beyaert R Afonina IS. Polo-like kinase 1 (Plk1) signaling in Cancer and beyond. Biochem Pharmacol. (2021) 193:114747. doi: 10.1016/j.bcp.2021.114747, PMID: 34454931

[72] Su S Chhabra G Singh CK Ndiaye MA Ahmad N. Plk1 inhibition-based combination therapies for Cancer management. Transl Oncol. (2022) 16:101332. Epub 2022/01/02. doi: 10.1016/j.tranon.2021.101332, PMID: 34973570 PMC8728518

[73] Yu Z Deng P Chen Y Liu S Chen J Yang Z. Inhibition of the Plk1-coupled cell cycle machinery overcomes resistance to Oxaliplatin in colorectal Cancer. Adv Sci (Weinh). (2021) 8:e2100759. doi: 10.1002/advs.202100759, PMID: 34881526 PMC8655181

[74] Taherdangkoo K Nezhad SRK Hajjari M. Mir-485-3p suppresses colorectal Cancer Via targeting Tpx2. Bratisl Lek Listy. (2020) 121:302–7. doi: 10.4149/BLL_2020_048, PMID: 32356447

[75] Zhang Q Di J Ji Z Mi A Li Q Du X. Kif20a predicts poor survival of patients and promotes colorectal Cancer tumor progression through the Jak/Stat3 signaling pathway. Dis Markers. (2020) 2020:1–11. doi: 10.1155/2020/2032679, PMID: 32695240 PMC7368235

[76] Kang DH Woo J Kim H Kim SY Ji S Jaygal G. Prognostic relevance of Hjurp expression in patients with surgically resected colorectal Cancer. Int J Mol Sci. (2020) 21:7928. doi: 10.3390/ijms21217928, PMID: 33114545 PMC7662712

[77] Liang Y-C Su Q Liu Y-J Xiao H Yin H-Z. Centromere protein a (Cenpa) regulates metabolic reprogramming in the Colon Cancer cells by transcriptionally activating Karyopherin subunit alpha 2 (Kpna2). Am J Pathol. (2021) 191:2117–32. doi: 10.1016/j.ajpath.2021.08.010, PMID: 34508688

[78] Zhang H Luo Z Tang J Tian J Xiao Y Sun C. Transcription factor Nfic functions as a tumor suppressor in lung squamous cell carcinoma progression by modulating Lncrna Casc2. Cell Cycle. (2022) 21:63–73. doi: 10.1080/15384101.2021.1995130, PMID: 34985387 PMC8837250

[79] Katerndahl CDS Rogers ORS Day RB Cai MA Rooney TP Helton NM. Tumor suppressor function of Gata2 in acute Promyelocytic leukemia. Blood. (2021) 138:1148–61. doi: 10.1182/blood.2021011758 34125173 PMC8570055

[80] Hays E Bonavida B. Yy1 regulates Cancer cell immune resistance by modulating Pd-L1 expression. Drug Resist Updat. (2019) 43:10–28. doi: 10.1016/j.drup.2019.04.001, PMID: 31005030

[81] Han F Zhang L Liao S Zhang Y Qian L Hou F. The interaction between S100a2 and Kpna2 mediates Nfya nuclear import and is a novel therapeutic target for colorectal Cancer metastasis. Oncogene. (2022) 41:657–70. doi: 10.1038/s41388-021-02116-6, PMID: 34802034

[82] Fang Z Lin M Chen S Liu H Zhu M Hu Y. E2f1 promotes cell cycle progression by stabilizing spindle Fiber in colorectal Cancer cells. Cell Mol Biol Lett. (2022) 27:90. doi: 10.1186/s11658-022-00392-y, PMID: 36221072 PMC9552509

[83] Li Q Zhang L You W Xu J Dai J Hua D. Prdm1/Blimp1 induces Cancer immune evasion by modulating the Usp22-Spi 1-Pd-L1 Axis in hepatocellular carcinoma cells. Nat Commun. (2022) 13:7677. doi: 10.1038/s41467-022-35469-x 36509766 PMC9744896

[84] Xiao Q Gan Y Li Y Fan L Liu J Lu P. Mef2a transcriptionally Upregulates the expression of Zeb2 and Ctnnb1 in colorectal Cancer to promote tumor progression. Oncogene. (2021) 40:3364–77. doi: 10.1038/s41388-021-01774-w, PMID: 33863999 PMC8116210

[85] Li LY Yang Q Jiang YY Yang W Jiang Y Li X. Interplay and cooperation between Srebf1 and master transcription factors regulate lipid metabolism and tumor-promoting pathways in squamous Cancer. Nat Commun. (2021) 12:4362. Epub 2021/07/18. doi: 10.1038/s41467-021-24656-x, PMID: 34272396 PMC8285542

[86] Choi KH Shin CH Lee WJ Ji H Kim HH. Dual-Strand tumor suppressor Mir-193b-3p and-5p inhibit malignant phenotypes of lung Cancer by suppressing their common targets. Biosci Rep. (2019) 39:BSR20190634. doi: 10.1042/BSR20190634, PMID: 31262974 PMC6630026

[87] Toolabi N Daliri FS Mokhlesi A Talkhabi M. Identification of key regulators associated with Colon Cancer prognosis and pathogenesis. J Cell Commun Signal. (2022) 16:115–27. Epub 2021/03/27. doi: 10.1007/s12079-021-00612-8, PMID: 33770351 PMC8688655

[88] Ghafouri-Fard S Khoshbakht T Hussen BM Abdullah ST Taheri M Samadian M. A review on the role of Mir-16-5p in the carcinogenesis. Cancer Cell Int. (2022) 22:342. doi: 10.1186/s12935-022-02754-0, PMID: 36348403 PMC9644515

[89] Li C Wang R Wu A Yuan T Song K Bai Y. Sars-Cov-2 as potential Microrna sponge in Covid-19 patients. BMC Med Genet. (2022) 15:94. doi: 10.1186/s12920-022-01243-7, PMID: 35461273 PMC9034446

[90] Rodriguez A Vigorito E Clare S Warren MV Couttet P Soond DR. Requirement of Bic/Microrna-155 for Normal immune function. Science. (2007) 316:608–11. doi: 10.1126/science.1139253, PMID: 17463290 PMC2610435

[91] Sacar Demirci MD Adan A. Computational analysis of Microrna-mediated interactions in Sars-Cov-2 infection. PeerJ. (2020) 8:e9369. doi: 10.7717/peerj.9369, PMID: 32547891 PMC7278893

[92] Nemani K Li C Olfson M Blessing EM Razavian N Chen J. Association of Psychiatric Disorders with mortality among patients with Covid-19. JAMA Psychiat. (2021) 78:380–6. doi: 10.1001/jamapsychiatry.2020.4442 PMC7841576 33502436

[93] Jansen Van Vuren E Steyn SF Brink CB Möller M Viljoen FP Harvey BH. The neuropsychiatric manifestations of Covid-19: interactions with Psy Chiatric illness and pharmacological treatment. Biomed Pharmacother. (2021) 135:111200. doi: 10.1016/j.biopha.2020.111200 33421734 PMC7834135

[94] Coughlin SS. Anxiety and depression: linkages with viral diseases. Public Health Rev. (2012) 34:1–17. doi: 10.1007/BF03391675 25264396 PMC4175921

[95] Babino G Argenziano G Balato A. Impact in contact dermatitis during and after Sars-Cov2 pandemic. Curr Treat Options Allergy. (2022) 9:19–26. doi: 10.1007/s40521-022-00298-2 35194543 PMC8830973

[96] Grivas P Khaki AR Wise-Draper TM French B Hennessy C Hsu CY. Association of Clinical Factors and Recent Anticancer Therapy with Covid-19 severity among patients with Cancer: a report from the Covid-19 and Cancer consortium. Ann Oncol. (2021) 32:787–800. doi: 10.1016/j.annonc.2021.02.024, PMID: 33746047 PMC7972830

[97] Paul SM Pinna G Guidotti A. Allopregnanolone: from molecular pathophysiology to therapeutics. Hist Perspect Neurobiol Stress. (2020) 12:100215. doi: 10.1016/j.ynstr.2020.100215, PMID: 32435665 PMC7231972

[98] Son HJ Sohn SH Kim N Lee H-N Lee SM Nam RH. Effect of estradiol in an Azoxymethane/dextran sulfate sodium-treated mouse model of colorectal Cancer: implication for sex difference in colorectal Cancer development. Cancer Res Treat. (2019) 51:632–48. doi: 10.4143/crt.2018.060, PMID: 30064198 PMC6473282

[99] Zhang Y-L Wen X-D Guo X Huang S-Q Wang T-T Zhou P-T. Progesterone suppresses the progression of colonic carcinoma by increasing the activity of the Gadd45α/Jnk/C-Jun Signalling pathway. Oncol Rep. (2021) 45:1–13. doi: 10.3892/or.2021.8046 33846816 PMC8054317

[100] Ramírez-de-Arellano A Gutiérrez-Franco J Sierra-Diaz E Pereira-Suárez AL. The role of estradiol in the immune response against Covid-19. Hormones. (2021) 20:657–67. doi: 10.1007/s42000-021-00300-7, PMID: 34142358 PMC8210971

[101] Bilezikian JP Bikle D Hewison M Lazaretti-Castro M Formenti AM Gupta A. Mechanisms in endocrinology: vitamin D and Covid-19. Eur J Endocrinol. (2020) 183:R133–47. doi: 10.1530/EJE-20-0665, PMID: 32755992 PMC9494342

[102] Huang C-Y Weng Y-T Li P-C Hsieh N-T Li C-I Liu H-S. Calcitriol suppresses Warburg effect and cell growth in human Colorect Al Cancer cells. Life (Basel). (2021) 11:963. doi: 10.3390/life11090963 34575112 PMC8466965

[103] Elamir YM Amir H Lim S Rana YP Lopez CG Feliciano NV. A randomized pilot study using Calcitriol in hospitalized Covid-19 patients. Bone. (2022) 154:116175. doi: 10.1016/j.bone.2021.116175, PMID: 34508882 PMC8425676

[104] Liu J Zhang L Gao J Zhang B Liu X Yang N. Discovery of Genistein derivatives as potential Sars-Cov-2 Main protease inhibitors by virtual screening, molecular dynamics simulations and Admet analysis. Front Pharmacol. (2022) 13:961154. doi: 10.3389/fphar.2022.961154 36091808 PMC9452787

[105] Park C Cha H-J Lee H Hwang-Bo H Ji SY Kim MY. Induction of G2/M cell cycle arrest and apoptosis by Genistein in human bladder Cancer T24 cells through inhibition of the Ros-dependent Pi3k/Akt signal transduction pathway. Antioxidants. (2019) 8:327. doi: 10.3390/antiox8090327, PMID: 31438633 PMC6769882

[106] Uckun F Evans W Forsyth C Waddick K Tuel-Ahlgren L Chelstrom L. Biotherapy of B-cell precursor leukemia by targeting Genistein to Cd19-associated tyrosine kinases. Science. (1995) 267:886–91. doi: 10.1126/science.7531365, PMID: 7531365

[107] Liu X Lan Y Zhang L Ye X Shen Q Mo G. Genistein exerts anti-colorectal Cancer actions: clinical reports, com Putational and validated findings. Aging (Albany NY). (2023) 15:3678–89. doi: 10.18632/aging.204702 37155147 PMC10449307

[108] Huang H Liao D Liang L Song L Zhao W. Genistein inhibits rotavirus replication and Upregulates Aqp4 expression in rotavirus-infected Caco-2 cells. Arch Virol. (2015) 160:1421–33. doi: 10.1007/s00705-015-2404-4, PMID: 25877820

[109] Ma S Song W Xu Y Si X Zhang D Lv S. Neutralizing tumor-promoting inflammation with polypeptide-dexamethasone conjugate for microenvironment modulation and colorectal cancer therapy. Biomaterials. (2020) 232:119676. doi: 10.1016/j.biomaterials.2019.119676 31896516

[110] Namazi N. The effectiveness of dexamethasone as a combination therapy for Covid-19. Acta Pharma. (2022) 72:345–58. doi: 10.2478/acph-2022-0030, PMID: 36651541

[111] Zhang Z Ji Y Hu N Yu Q Zhang X Li J. Ferroptosis-induced anticancer effect of resveratrol with a biomimetic Nano-delivery system in colorectal Cancer treatment. Asian J Pharm Sci. (2022) 17:751–66. doi: 10.1016/j.ajps.2022.07.006 36382309 PMC9640689

[112] Wester RA van Voorthuijsen L Neikes HK Dijkstra JJ Lamers LA Frölich S. Retinoic acid signaling drives differentiation toward the absorptive L Ineage in colorectal cancer. iScience. (2021) 24:103444. doi: 10.1016/j.isci.2021.103444 34877501 PMC8633980

[113] McCreary MR Schnell PM Rhoda DA. Randomized double-blind placebo-controlled proof-of-concept trial of resveratrol for outpatient treatment of mild coronavirus disease (Covid-19). Sci Rep. (2022) 12:10978. doi: 10.1038/s41598-022-13920-9, PMID: 35768453 PMC9243086

[114] Sarohan AR. Covid-19: endogenous retinoic acid theory and retinoic acid depletion syndrome. Med Hypotheses. (2020) 144:110250. doi: 10.1016/j.mehy.2020.110250, PMID: 33254555 PMC7481114

